# Mechanical Milling: A Superior Nanotechnological Tool for Fabrication of Nanocrystalline and Nanocomposite Materials

**DOI:** 10.3390/nano11102484

**Published:** 2021-09-24

**Authors:** M. Sherif El-Eskandarany, Abdulsalam Al-Hazza, Latifa A. Al-Hajji, Naser Ali, Ahmed A. Al-Duweesh, Mohammad Banyan, Fahad Al-Ajmi

**Affiliations:** Nanotechnology and Applications Program, Energy and Building Research Center, Kuwait Institute for Scientific Research, Safat 13109, Kuwait; ahazza@kisr.edu.kw (A.A.-H.); lhajji@kisr.edu.kw (L.A.A.-H.); nmali@kisr.edu.kw (N.A.); aduweesh@kisr.edu.kw (A.A.A.-D.); mbanyan@kisr.edu.kw (M.B.); ftajmi@kisr.edu.kw (F.A.-A.)

**Keywords:** nanomaterials, powder technology, solid-state reaction, nanocomposite materials, hard nanomaterials, gas-solid reaction, reactive ball milling

## Abstract

Throughout human history, any society’s capacity to fabricate and refine new materials to satisfy its demands has resulted in advances to its performance and worldwide standing. Life in the twenty-first century cannot be predicated on tiny groupings of materials; rather, it must be predicated on huge families of novel elements dubbed “advanced materials”. While there are several approaches and strategies for fabricating advanced materials, mechanical milling (MM) and mechanochemistry have garnered much interest and consideration as novel ways for synthesizing a diverse range of new materials that cannot be synthesized by conventional means. Equilibrium, nonequilibrium, and nanocomposite materials can be easily obtained by MM. This review article has been addressed in part to present a brief history of ball milling’s application in the manufacture of a diverse variety of complex and innovative materials during the last 50 years. Furthermore, the mechanism of the MM process will be discussed, as well as the factors affecting the milling process. Typical examples of some systems developed at the Nanotechnology and Applications Program of the Kuwait Institute for Scientific Research during the last five years will be presented in this articles. Nanodiamonds, nanocrystalline hard materials (e.g., WC), metal-matrix and ceramic matrix nanocomposites, and nanocrystalline titanium nitride will be presented and discussed. The authors hope that the article will benefit readers and act as a primer for engineers and researchers beginning on material production projects using mechanical milling.

## 1. Introduction

Despite the fact that conventional material categories (metals and metal alloys, ceramics, polymers, and composites) are incapable of meeting the demands of contemporary modern industries, a newcomer known as “advanced materials” has carved out a major niche in material functional classifications. Advanced materials, on the other hand, may be characterized by a number of techniques, depending on their properties and uses. They are materials that outperform conventional materials and are used to fabricate high-tech goods [1].

### Strategies for the Development of New Material Categories

Nonetheless, while there are several fabrication techniques, which can be employed for producing conventional materials via hydrometallurgy, pyrometallurgy, and powder metallurgy approaches, none of these techniques is capable of readily preparing advanced materials. Over the past six decades, materials scientists have devised a number of synthesis processes and methods for the synthesizing of new material families, often referred to as advanced or “high-tech” materials, with distinguishing chemical, physical, and mechanical properties [2]. These novel techniques for processing and fabricating materials have enabled scientists to alter the subatomic structure of materials and tailor them to a desired and predetermined structure. To achieve high performance characterization, altering the structure of the materials (e.g., long- or short-range ordered) has an impact on the material’s overall properties. Additionally, modifying the morphological and microscopic characteristics of materials results in noticeable changes in their properties and behaviors [3,4]. It may be inferred that the way a material is prepared (material processing and fabrication) has an influence on its atomic arrangements and microscopic characteristics, which affects not only the product’s overall attributes, but also its performance and future uses [5].

Over the last few decades, materials scientists’ research has enabled us to produce a diverse spectrum of complex materials utilizing innovative preparation processes. The fabrication techniques for advanced materials can be broadly classified as follows: Mechanically aided approaches include the following: (1) mechanically driven solid-state reaction approaches; (2) thermally assisted approaches; (3) mechanically assisted approaches; (4) chemically assisted approaches; (5) lithographic approaches; (6) vapor deposition approaches; and (7) liquid-phase fabrication approaches [6]. Ball milling, rapid solidification, atomization, sputtering, chemical vapor deposition, electron beam physical vapor deposition, arc discharge, laser ablation, photolithography, nano-imprint lithography, sol-gel, atomic force microscope nanostencil, plasma enhanced chemical vapor deposition, plasma enhanced chemical vapor deposition, and atomic layer deposition are some examples of the preparation methods that are used to fabricate new materials [1].

## 2. Mechanically-Induced Milling for Preparing Advanced Materials

Powder metallurgy (P/M) is a manufacturing process used for the production of cost-effective tools and efficient components made from metallic, ceramic, or composite powders. In fact, P/M is not a novel material science process; it dates all the way back to 3000 B.C. when the Egyptians employed it to make iron powder from “sponge iron” for their implements [7]. The P/M technique has been recognized as a viable way for forming high melting point metals, metal oxides, and cemented carbides in net or near-net shape without melting or casting castings [8]. Throughout World War II and into the 1960s, a variety of novel composites, ferrous and nonferrous-based materials were created. In the last 50 years, enormous progress has been achieved in the field of powder consolidation, with the invention of novel powder pressing techniques such as cold/hot isostatic pressing, spark plasma sintering, shock-wave consolidation, and induction hot pressing [9,10].

**Figure 1:**
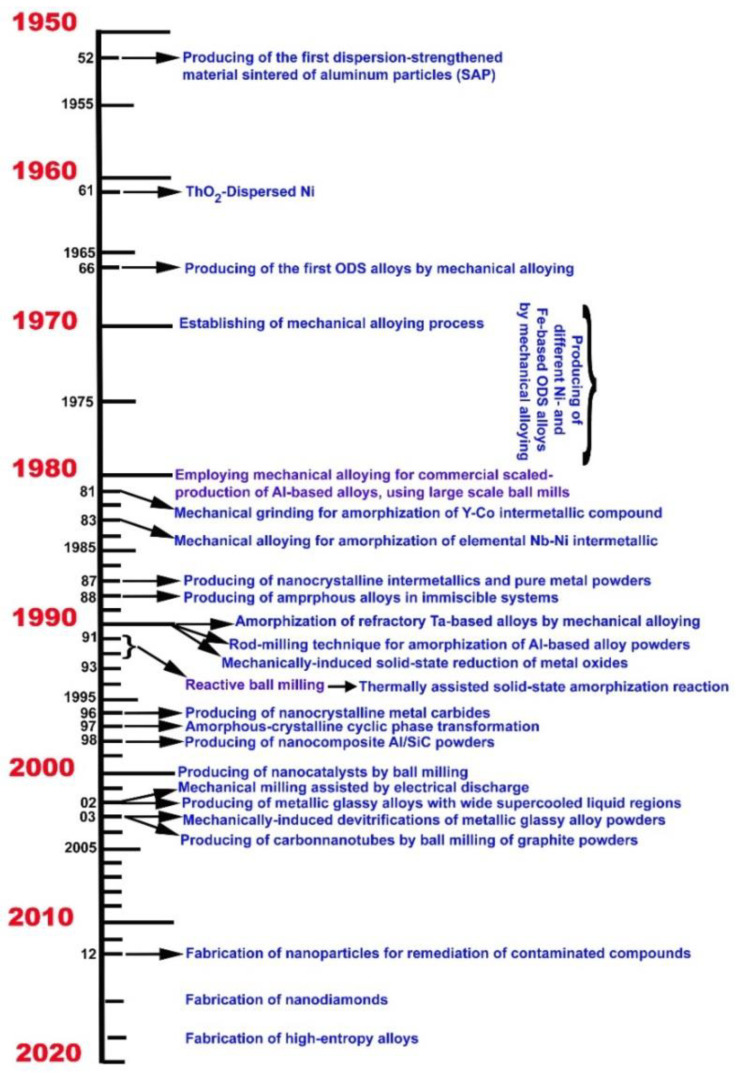
A timeline presentation displaying the evolution of mechanical alloying since 1950, along with some instances of novel materials obtained by this process.

Milling is the process of reducing relatively coarse materials to a desired fineness [1]. This process is a common routine approach of comminuting procedure used in mineral dressing to separate precious mineral grains from mother rock by rotating cylindrical steel vessels holding a charge of free-moving balls within the mill [11]. Mechanical alloying (MA) [12] is a typical solid-state process that occurs at room temperature between powders of diffusion couples utilizing various types of ball mills. Ball milling has evolved over the last five decades from a standard technique in mineral dressing and powder metallurgy, where it was primarily used for particle size reduction and powder blending, to its current prominence as a powerful method for the preparation of materials with improved physical and mechanical properties, as well as new phases and engineering materials. MA is widely regarded as one of the most efficient nanotechnology techniques for the top-down preparation approach of a broad variety of nanocrystalline, nanoparticles, and nanocomposites materials. As a result, the term MA is becoming increasingly prevalent in the materials science, metallurgy, and nanotechnology literatures [13]. The MA method, which incorporates several types of milling equipment [14], has garnered much attention as a powerful tool for producing a range of advanced materials. Figure 1 depicts a schematic history of the evolution of the mechanical alloying process since 1950.

Benjamin, dubbed “The Godfather of MA,” invented the MA process in the 1960s at The International Nickel Company (INCO) as part of a program to develop a material that combines oxide dispersion strengthening and gamma prime precipitation hardening in nickel-based superalloys for gas turbine applications [15]. The phrase “mechanical alloying” was introduced by Ewan C. MacQueen in a patent owned by INCO in the United States [12]. This innovative approach produced fine and consistent dispersions of metal oxide particles (Al_2_O_3_, Y_2_O_3_, and ThO_2_) in nickel-base superalloys [15].

In mid-1966, researchers investigated the possibility of employing the ball-milling method some useful alloys powder technology. The ability of this method to coat the hard-ceramics phase (e.g., WC or ZrO_2_) with a soft-metallic phase (Co or Ni) was also proposed [16]. This choice was selected because the ball-milling process is capable of preparing hard composite-based materials, which can be employed for coating purposes. It is well established that when metallic particles are ball milled, both welding and fracturing occur, and that high energy ball mills may significantly expedite the grinding and fracturing processes. Additionally, the thermodynamic activity of reactive gamma prime producing elements such as aluminum and titanium might be lowered by orders of magnitude by milling them with less reactive metals (e.g., Ni), rather than relying on fairly costly atomized pre-alloy powders [17].

Apart from fabricating ODS alloys using the ball-milling approach and then beneficiating them, Koch et al. [18] demonstrated the first novel technique for synthesizing Ni_60_Nb_40_ amorphous alloy via high energy mixer milling of elemental Ni and Nb powders. Since then, the MA method has been utilized to successfully fabricate a large variety of amorphous alloys.

### 2.1. The Necessity of MA

Since the first pioneering investigation introduced by Koch [18], the MA technique has emerged as a desirable and powerful method for producing different amorphous alloy systems. Furthermore, MA has introduced a practical solution as a solid-state process to overcome the difficulties of preparing a number of alloys that aren’t possible to prepare with a different technique, such as the Al-Ta [19] and Al-Nb [20] binary systems that reveal significant variation in the melting points, taking the Al-Ta system as an example (melting points of Al and Ta are 661 °C and 3020 °C, respectively) [21]. It also demonstrates a high capability for the preparation of immiscible systems (e.g., Cu-Ta [22]).

### 2.2. MA and Mechanical Disordering (MD)

**Figure 2:**
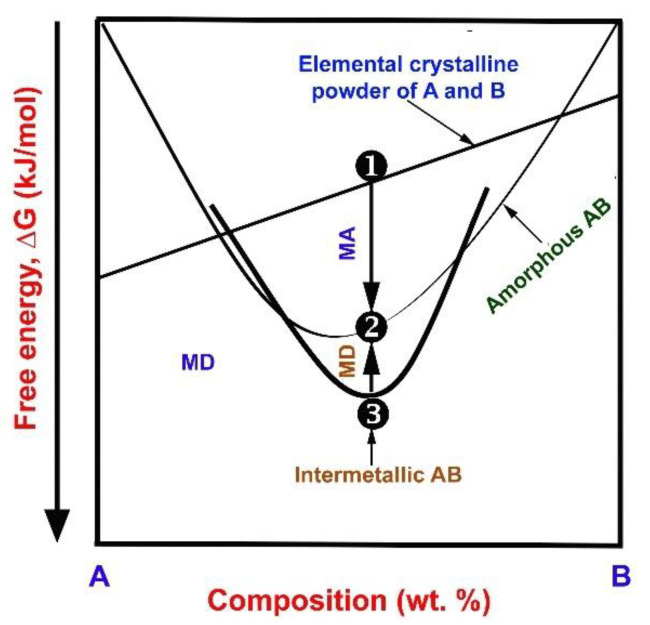
Diagrammatic illustration of the free energy (DG) for the phases engaged in the mechanical alloying and mechanical disordering processes, commencing with (1) a mixture of two elemental powders (A + B), and (2) an intermetallic phase of AB exposed to the MA and MD processes, respectively.

Based on the free energy change that occurs when starting powders are subjected to a high energy milling process, the process can be classified into two categories: (1) mechanical alloying (MA), and (2) mechanical disordering (MD) or mechanical activation [23]. As previously stated, the MA process is a method where the elemental metal powders are repeatedly fracturing and rewelding in a high energy ball mill. In the MD process, however, the starting feedstock materials are intermetallic alloys, so that these crystalline alloys tend to convert into an amorphous solid state by relaxing the short-range order without compositional alterations [24]. Figure 2 depicts a free-energy (DG) diagram of two phases engaged in the MA and MD processes. In these two opposite direction processes, it can be realized that the starting powders (A + B) for MA and MD (AB) are mixture of two elemental powders (Point 1 in Figure 2), and intermetallic phase (Point 3 in Figure 2), respectively. Suzuki [24] pointed out that the two processes are thermodynamically incompatible reactions. As previously stated, the MA method produces metastable amorphous alloy powders by mechanically driven solid-state them with elemental crystalline powders. Because the starting state is just a mixture of pure A and B crystals, its free energy can be placed along the straight line connecting the DG of pure A and B elemental powders. In contrast to Point 1, Point 2 refers to the free energy of the end product (amorphous phase); a-AB. By comparison, Point 3 represents the DG of the same composition for crystalline intermetallic phase. The MD process is carried out at an ambient temperature that is high enough to conduct phase transformation into the same amorphous phase without compositional change [23,24].

### 2.3. Types of Ball Mill

Although there are various kinds of ball mills, including drum ball mills, jet ball mills, bead mills, horizontal rotary ball mills, vibration ball mills, and planetary ball mills, they can be divided into two categories based on their rotation speed: high energy ball mills, and low energy ball mills. In fact, the type of ball mill used is determined by the milling goals.

#### 2.3.1. High Energy Ball Mills

- Attritor Ball Mill

**Figure 3:**
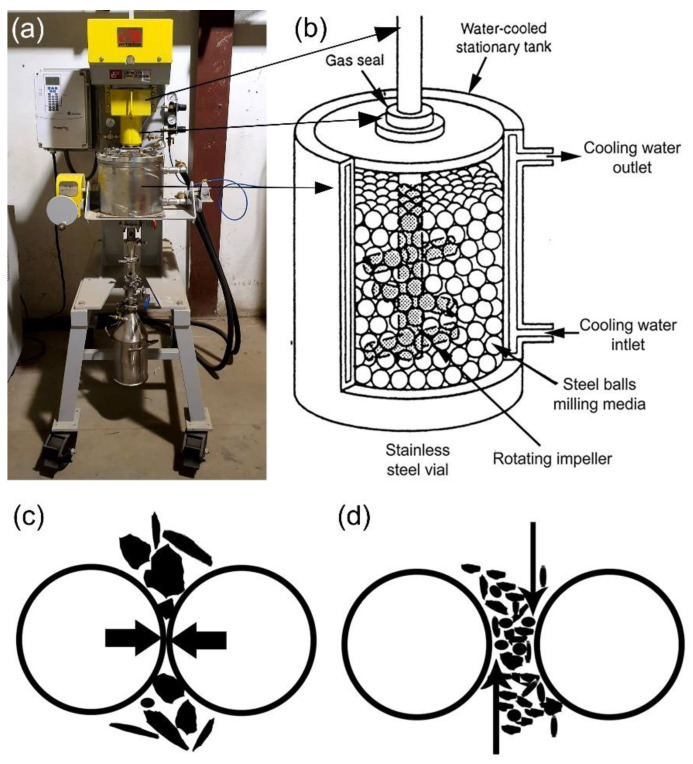
(**a**) A Photo of an attritor ball mill; sketches illustrate (**b**) ball movement inside attritor ball mill, (**c**) impact, and (**d**) shear forces generated by the ball milling media. The equipment is housed in the Nanotechnology and Applications Program (NAP), Energy and Building Research Center (EBRC), Kuwait Institute for Scientific Research (KISR).

The Attrition process is a simple and effective one, in which the starting materials are comminuted by means of free-moving beads that are set in motion by a stirrer (Figure 3a). Milling is accomplished by the agitation of an agitator that comprises a vertical rotating central shaft with horizontal arms referred to as impellers (Figure 3b) and rotates at a speed of 75 to 500 revolutions per minute.

Certain high-speed attrition mills operate at rates ranging from 400 to 2000 revolutions per minute. The milling media exert pressures on the milled powders via impact (Figure 3c) and shearing (Figure 3d).

- Shaker Mills

**Figure 4:**
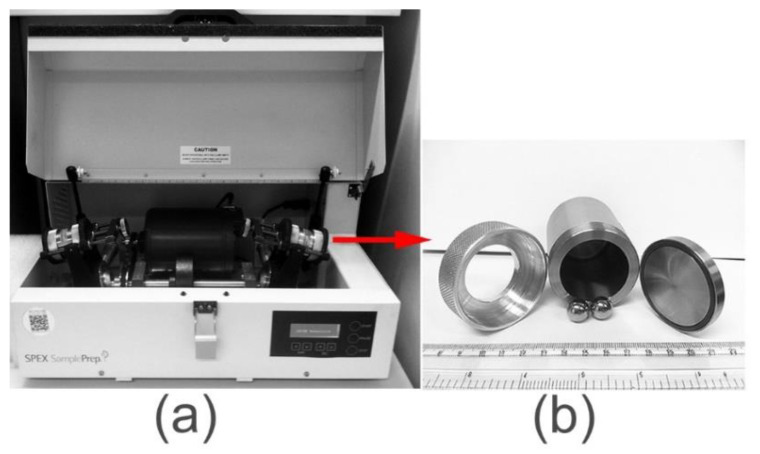
Photos of (**a**) SPEX8000D high energy ball mill. The milling tools (vials and balls) used of this type of ball mills are shown in (**b**). The equipment is housed in the NAP, EBRC of KISR.

SPEX is one brand of shaker mill (SPEX CertPrep, Inc., Metuchen, NJ, USA). The manufacturer offers two models: the Spex 8000M (Figure 4a) and the Spex 8000D. The first model suspends a single vial in an arm that oscillates rapidly along multiple axes, whereas the second model suspends two vials in two arms. At around 1200 rpm, the mill agitates the powder through the balls’ motions in the three perpendicular directions.

- Planetary Mall Mills

**Figure 5:**
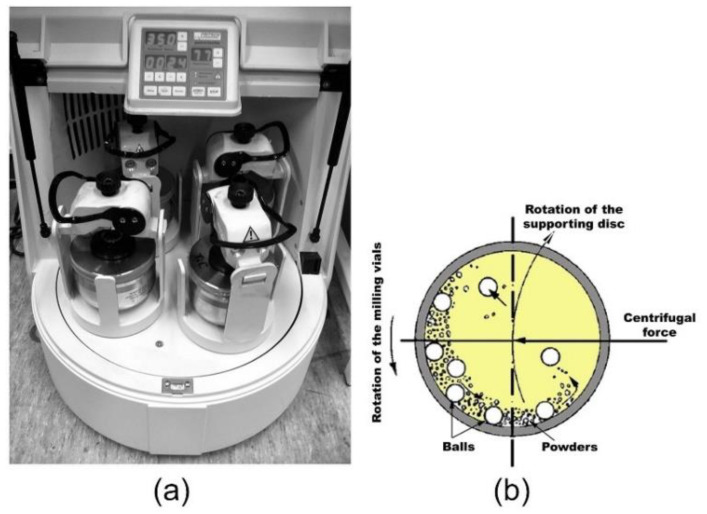
(**a**) Photograph of the Fritsch Planetary-type high energy ball mill (Pulverisette P5). The movement of the balls during the milling process is schematically depicted in (**b**). The equipment is housed in three locations: NAP, EBRC, and KISR.

Planetary Ball Mills (Figure 5a) are commonly utilized mills in MA and MD processes for synthesizing nearly all types of metastable, and composite materials. Because the milling stock and balls exit via the inside wall of the vial (milling bowl or vial), and the effective centrifugal force (Figure 5b) in this type of mill may reach up to twenty times the gravitational acceleration, the milling media carries a tremendous amount of energy. The milling charge is propelled forward by centrifugal forces generated by the rotating supporting disc and the vial’s self-turning action (balls and powder). Because the supporting disc and the vial have opposing rotating orientations, the centrifugal forces are alternately coordinated and resisted. As a consequence, the grinding medium and charged powders alternately roll on the inner wall of the vial before being lifted and thrown across the bowl at high velocity, as schematically illustrated in Figure 5b.

#### 2.3.2. Low Energy Ball Mills

- Tumbler Ball Mill

**Figure 6:**
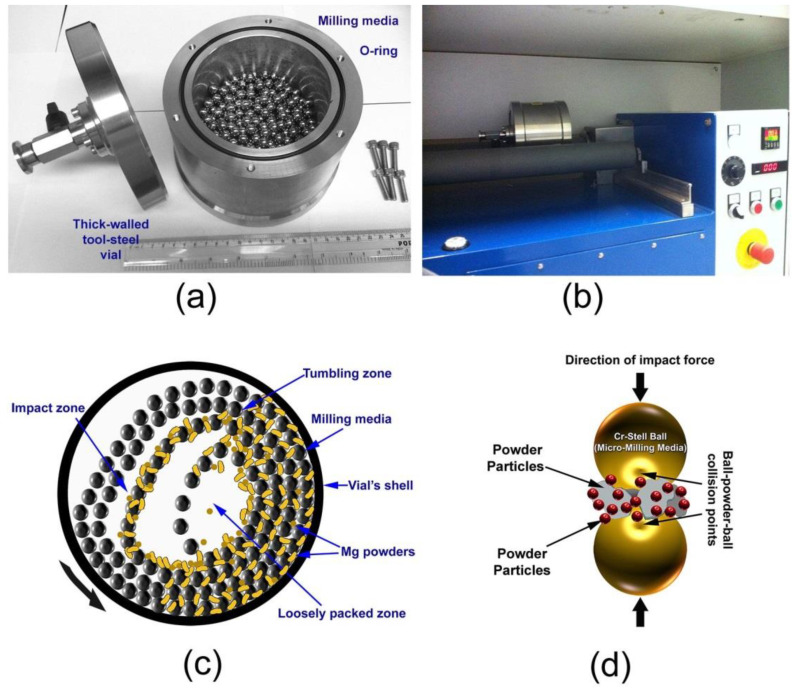
Photographs from NAP, EBRC, and KISR demonstrate a laboratory-scale 1 L roller mill setup. (**a**) The milling tools, which include the balls and vial, (**b**) The ball milling process, which utilizes a roller mill, (**c**) Schematic representations of ball positions and movement inside the vial of a tumbler mall mill operating in dynamic mode, and (**d**) A typical ball-powder-ball collusion during a low energy tumbling process.

The useful kinetic energy can be transferred to the powder particles of the reactant materials in these types of mills (Figure 6a,b) via (2) collisions between the balls and the powders (Figure 6c,d); (2) pressure loading of powders pinned between milling media or between the milling media and the liner (Figure 6c); (3) impact of the falling milling media, shear, and abrasion caused by draping (Figure 6c). Ball mill tumblers have been used to successfully produce a variety of mechanically alloyed powders. (See, for example, Ref. [1]). However, while this type of low energy mill may increase the amount of time necessary to complete the MA process, it generates homogenous and uniform powders [1]. Additionally, it is less costly than systems that use a lot of energy. Additionally, tumbling mills are low-maintenance and easy to run (Figure 6b).

#### 2.3.3. Factors Affecting the Milling Process

**Figure 7:**
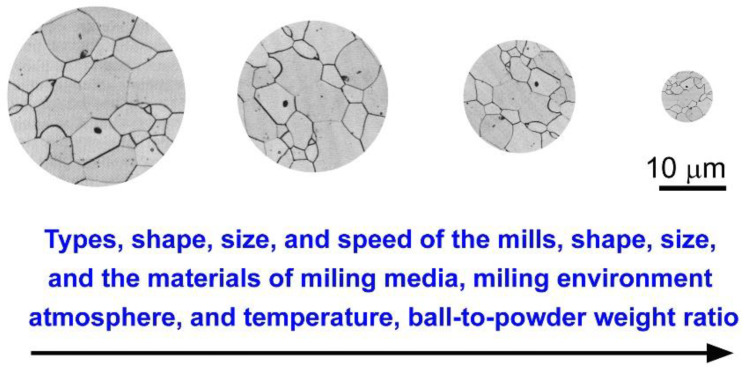
Factors impacting the milling process and affecting the particle size distribution of the powders.

Powder milling using the MA, MD, or MM processes, like any other method for synthesizing materials, is impacted by a variety of parameters that are crucial in the manufacture of homogeneous ultrafine powders (Figure 7). It is well-known that milling conditions have a direct effect on the properties of the final product’s milled powders, including particle size distribution, degree of disorder or amorphization, and final stoichiometry, and that the more comprehensive the control and monitoring of the milling conditions, the better the end product. Several critical factors affecting the milling technique include the following:

- Type of mills (low energy, high energy mills),
- Size of the milling vial,
- The materials of the milling tools (e.g., WC, steel alloys, ceramics)
- Shapes of milling media (balls or rods)
- Ball-to-powder weight ratio,
- Milling speed,
- Milling time,
- Milling atmosphere (inert gas or reactive gas such as hydrogen),
- Milling environment (dry or wet milling),
- Milling temperature,
- Impurities introduced to the powders upon using organic lubricant agents.

## 3. Nanocrystalline and Nanopowders Prepared by Ball Milling Technique at KISR

### 3.1. Background

Three types of nanomaterials exist: one-dimensional [1-D], two-dimensional [2-D], and three-dimensional [3-D]. The thickness of one-dimensional nanomaterials (e.g., thin films) should be nanoscale (1 to 100 nm). Nanotubes, nanorods, and nanowires are all examples of the second class of nanomaterials [2-D], which have two dimensions less than 100 nm in length and diameter. Nanoparticles, nanopowders, nanocrystalline powders, quantum dots, Bucky balls, and nanocrystals all fall under the third category of three-dimensional nanomaterials (3-D nanomaterials), which includes materials that are nanoscale in three dimensions.

In general, nanomaterials can be synthesized using one of two approaches: (i) top-down or (ii) bottom-up. A top–down approach is a strategy for fabricating nanoscaled structures/functional materials with the appropriate forms and properties by beginning with bigger dimensions and decreasing them to the required values [25]. Rolling, atomization, electrospinning, evaporation, laser ablation, RF-sputtering, and high energy sonication are all examples of conventional top-down nanofabrication processes. Notably, ball milling has been regarded as a cost-effective method for the top-down manufacture of a wide variety of nanoscaled powders [1].

In contrast to the top-down approach, the bottom-up approach employs sophisticated mechanisms and technologies to construct intricate nanoscale assemblies or controlled self-assembly from molecular or atomic components. CVD, atomic layer deposition (ALD), molecular beam epitaxy (MBE), gas phase condensation thermolysis, and dip-pen nanolithography (DPNL) are only a few of the bottom-up fabrication processes.

### 3.2. Nanodiamonds

Different processes can be used to create graphite-based nanomaterials such as fullerenes, graphene, carbon nanotubes, and nanodiamonds [26]. For instance, nanodiamonds, which were first synthesized by Bovenkerk et al. in 1959 [27], can be synthesized using a variety of techniques, including detonation [28], laser ablation [29], plasma-enhanced chemical vapor deposition (PECVD) [30], autoclave synthesis from supercritical fluids [31], carbide chlorination [32], graphite ion irradiation [33,34], ion irradiation of graphite, and a plasma process [33]. Carbon-carbon phase transformations, as well as the stability of nanodiamonds, have sparked considerable attention (see for example, Refs. [35,36,37,38]). At the macroscale, it was formerly believed that graphite was the most stable member of the carbon family at ambient temperatures and pressures, whereas diamond was considered a metastable carbon phase. As Barnard et al. [38], and Tyler et al. [39] proposed, it is now well established that nanoscaled nanodiamond particles (3–5 nm in diameter) are more stable than graphite. In these nanoparticles, the crystalline diamond core with perfect diamond lattice is invariably surrounded by an amorphous shell composed of sp^2^/sp^3^ links or an onion-like graphite shell [40].

Apart from the conventional methods for synthesizing nanodiamond materials, which necessitate the use of extremely high temperatures and/or pressures, there are other options [41,42,43,44]. Recently, El-Eskandarany published the first study demonstrating the feasibility of producing ultrafine nanodiamonds from commercial graphite powders using a high energy ball milling process at room temperature and normal pressure. [45]. In his work, 3 g of elemental graphite powders were balanced in a glove box with a high purity He gas atmosphere before being sealed with 40 Cr-steel balls (14 mm in diameter) at a 167:1 ball-to-powder weight ratio. The vial was then placed in a planetary-type high energy ball mill and rotated at 800 rpm for various ball milling times. Following selected milling runs, the as-milled powders were regularly released completely from the vial in the glove box. A new patch of graphite particles was charged and sealed inside the vial for subsequent milling cycles. To better understand the graphite-nanodiamond phase transitions, samples obtained at various phases of ball milling were studied using a variety of techniques.

**Figure 8:**
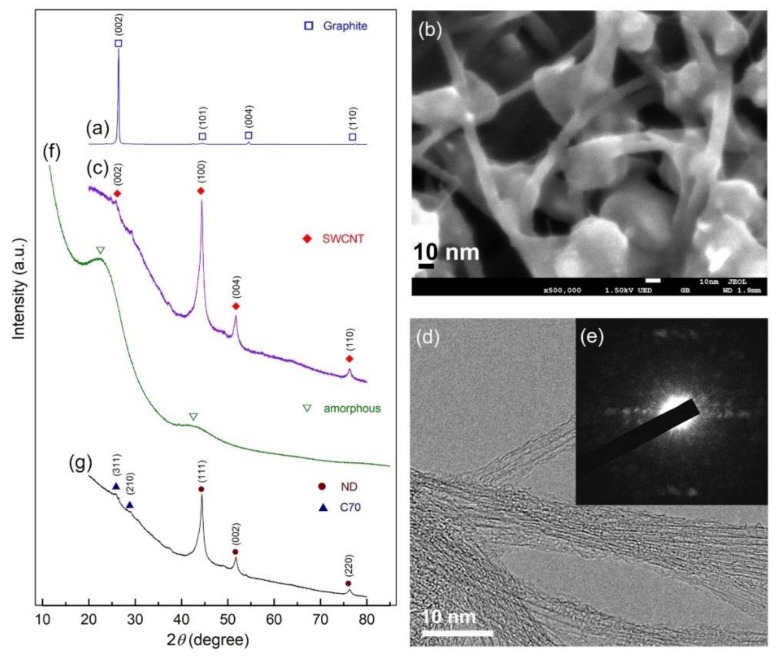
XRD diffraction patterns and FE-SEM images of the powders obtained after ball milling for (**a**) 0 h, and (**b**) 12 h, respectively. In (**c–e**), the XRD patterns and corresponding FE-HRTEM, NBDP of the sample obtained after 10 h are shown. The XRD patterns for the samples milled for 16 h and 28 h are displayed in (**f**,**g**), respectively. micrograph of graphite powders produced after 0 and 12 h of high energy ball milling, respectively.

We used both X-ray diffraction (XRD) and field emission high resolution electron microscopy (FE-HRTEM) techniques to track the progress of phase transitions during high energy ball milling of graphite powders at various milling speeds. The XRD patterns of the beginning graphite powders acquired during the early stages of ball milling (0–16 h) are shown in Figure 8. As illustrated in Figure 8a, the initial graphite (G) powders had large-scaled crystallites, as evidenced by the sharp Bragg peaks corresponding to hexagonal close packed, hcp- (002), -(101), -(004), and -(110). After 10 h of ball milling, the pattern displayed Bragg peaks indicative of single wall carbon nanotubes, SWCNs (PDF# 00-58-1638), as illustrated in Figure 8b [45]. The high-magnification field emission scanning electron microscope (FE-SEM) micrograph of the powders obtained after this stage of milling (12 h) showed the existence of untransformed G-nanoparticles (10–30 nm in diameter) attached to the surface of the micron-size aggregates SWCNTs (Figure 8b). Formation of metastable SWCNs was confirmed by XRD and HRTEM (Figure 8c,d) as well as the corresponding nano beam diffraction pattern, NBDP (Figure 8e) [45].

**Figure 9:**
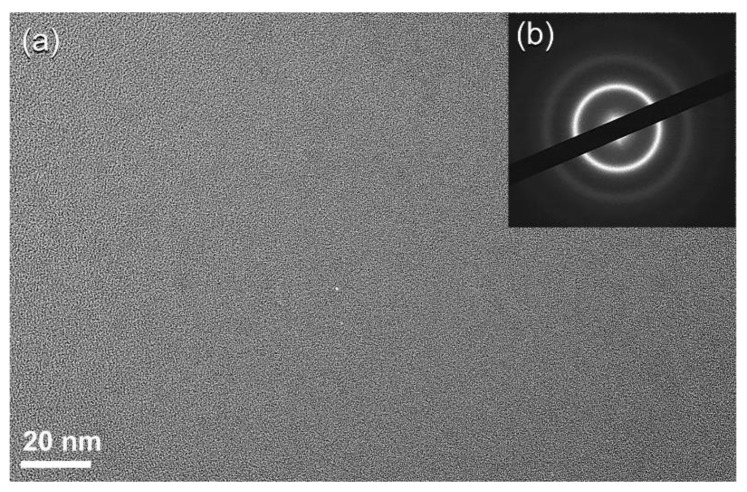
(**a**) FE-HRTEM image and (**b**) corresponding NBDP of the sample obtained after 16 h of milling.

After 16 h of ball milling, the crystalline peaks associated with hcp-SWCNTs vanished completely and were replaced with homogenous halo-diffuse peaks, the first and second maxima of which were identified at 22.56° and 42.94°, respectively (Figure 8f). This entails the transition of SWCNTs into amorphous carbon (a-C). HRTEM images corroborated the creation of the a-C phase, revealing a dense random-packing structure with a maze-like architecture, as illustrated in Figure 9a. Untransformed G-nanoparticles, SWCNTs, and/or nano-carbon fibers are barely visible in this image, implying a full SWCNTs to a-C phase change. Moreover, the NBDP associated with a selected zone in Figure 9b displays a halo-diffuse pattern of a typical amorphous phase (Figure 9b) [45].

**Figure 10:**
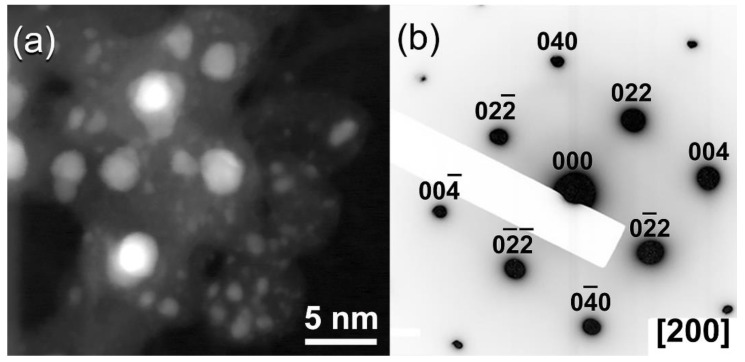
(**a**) Dark field image (DFI), and (**b**) corresponding NBDP of the sample obtained after 28 h of milling.

The dark field image (DFI) of the powders obtained after 28 h of ball milling is shown in Figure 10a. Obviously, the particles possessed spherical-like crystallinity with rather wide particle size distribution, ranging between 1.8 nm to 4.8 nm in diameter (Figure 10a). The NBDP of this fine particle is corresponding to NDs, as indexed by the zone axis (200) (Figure 10b) [45].

### 3.3. Tungsten Carbide

Among the hard materials, tungsten carbide (WC) has received considerable interest due to its unusual chemical and mechanical properties [46]. WC is an interstitial combination of carbon atoms filling a W crystal [46]. Since the early twentieth century, WC has been widely employed in the industry as cutting tool tips and wear-resistant parts. Due to its intrinsic resistance to oxidation and corrosion at elevated temperatures, WC is an excellent candidate for protective coatings for high-temperature electronics [47,48]. Unfortunately, nanocrystalline bulk WC materials are rarely used in industrial scale applications due to their limited sinterability even at elevated temperatures (1650–1900 °C) [49,50,51,52]. The possibility of improving mechanical properties including as hardness, elastic modulus, and fracture toughness has sparked interest in the manufacture of nanocrystalline WC ultrafine powders. Numerous methods, including chemical synthesis [46], mechanically induced solid state reduction [53], plasma-chemical interaction [54], and chemical vapor deposition (CVD), can be employed to synthesize polycrystalline superfine WC powders with an average grain size of 300–40 nm. However, mechanical milling (MM) is believed to be the most potent approach for generating nanocrystalline WC ultrafine powders on an industrial scale at near room temperature [51,53]. It has been demonstrated that ultrafine WC nanopowders with a spherical-like shape may be successfully generated employing WC milling equipment with a ball-to-powder weight ratio of 50:1 [51]. The milling procedure was carried out for 20 h at a speed of 400 rpm using a planetary-type ball mill.

**Figure 11:**
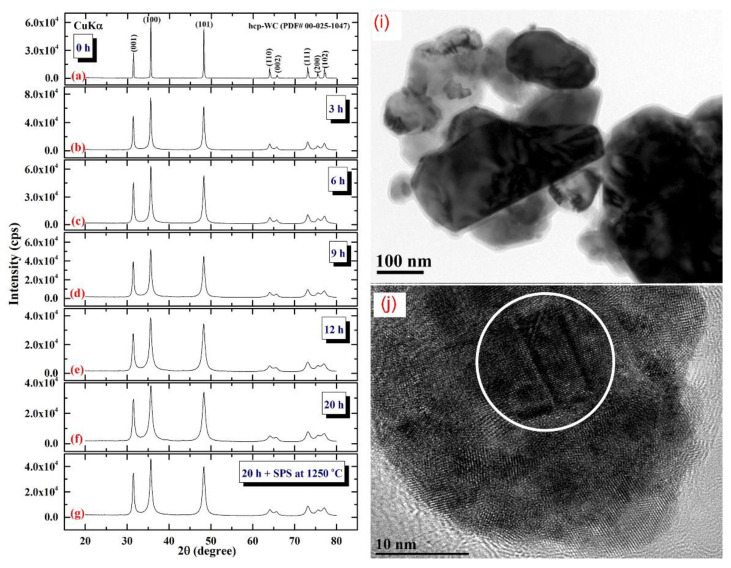
XRD patterns of ball milled WC powders treated for (**a**) 0, (**b**) 3, (**c**), 6 h, (**d**), 9 h, (**e**), 12, and (**f**), 20 h of milling. The powders produced after 20 h of milling and subsequently consolidated into bulk buttons at 1250 °C using the SPS method are depicted in the XRD (**g**). The bright field images (BFIs) of mechanically milled hcp-of-milling WC powders produced after 1 h and 3 h of high energy ball milling are shown in (**i**) and (**j**).

**Figure 12:**
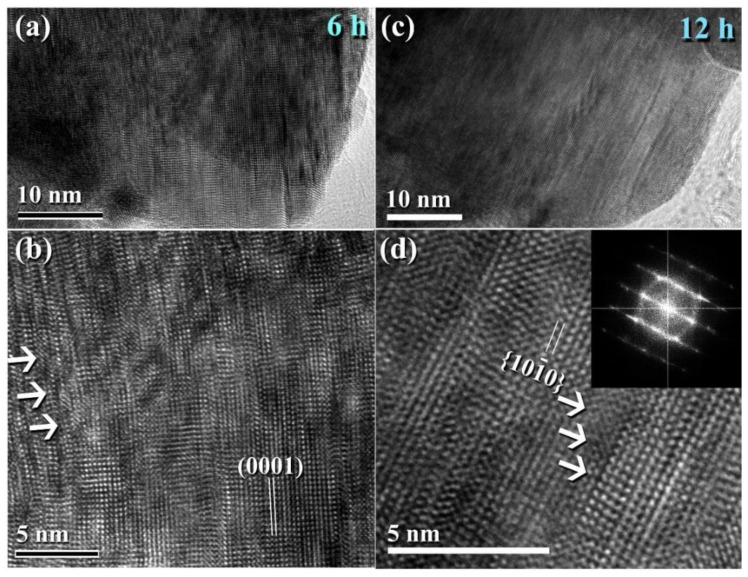
Lattice imperfections generated in WC crystals during the early stage of milling. HRTEM micrograph of ball milled WC powders after (**a**,**b**) 6 h, and (**c**,**d**) 12 h of milling.

Figure 11 illustrates the XRD patterns of mechanically milled pure hcp-WC powders obtained after varying ball milling times ranging from 0 to 20 h. (Figure 11a–g). Figure 11a shows that as-received powders (0 h) had significant Bragg peaks that were consistent with polycrystalline hcp-WC (PDF# 00-025-1047). This starting material powder had a bulky look and was composed of large particles with an uneven shape, ranging in diameter from 50 to 600 nanometers (Figure 11i). The Bragg peaks of powders milled for 3–6 h MM time exhibited substantial broadening as a result of the grain refining process caused by the milling action (Figure 11b,c). The powders formed after three hours of MM exhibited nano-twin lattice defects and stuck faults in their interior structure, as illustrated in Figure 11j. Additionally, the diameters of the powder particles obtained after three hours of MM were substantially reduced to approximately 28 nm (Figure 11j). Following this stage of MM, the powders comprise a variety of grains ranging in size from 8 nm to 10 nm in diameter. During the intermediate stage (6–12 h), additional broadening of the Bragg peaks was seen, together with a significant drop in their intensities (Figure 11d,e), implying continuing grain refining and the formation of finer particles. Moreover, the WC powders suffered from a great number of lattice imperfections, where clear stacking faults and dislocations existed inside the dark-contrast grains (Figure 12a).

The TEM micrograph of the bright-contrast region in Figure 12a reveals the presence of severe dislocations, which are denoted by the symbols in Figure 12b. These flaws were spread continually by increasing the MM time to 12 h (Figure 12c), as indicated by the obvious staking errors in Figure 12d. The plastic deformation of the WC crystal lattice that occurred during the early stages of MM was caused by slip and twinning, as illustrated in Figure 12a. This mechanical deformation is favored to be concentrated into the shear bands comprising a high density network of dislocations [55,56], as depicted in Figure 12b. At this stage of MM, the strain at the atomic level grows as the dislocation density increases.

**Figure 13:**
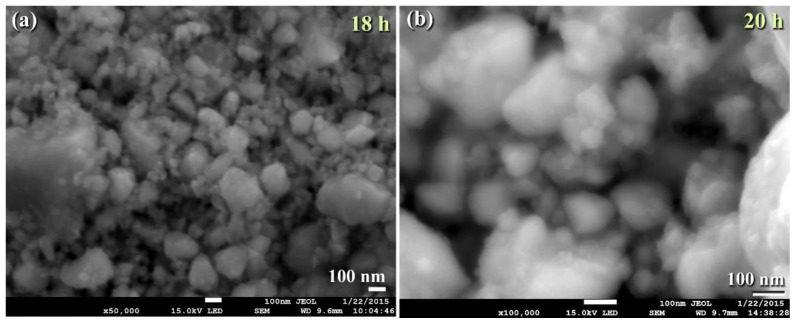
Micrographs FE-SEM ball milled WC powders after (**a**) 18, and (**b**) 20 h.

**Figure 14:**
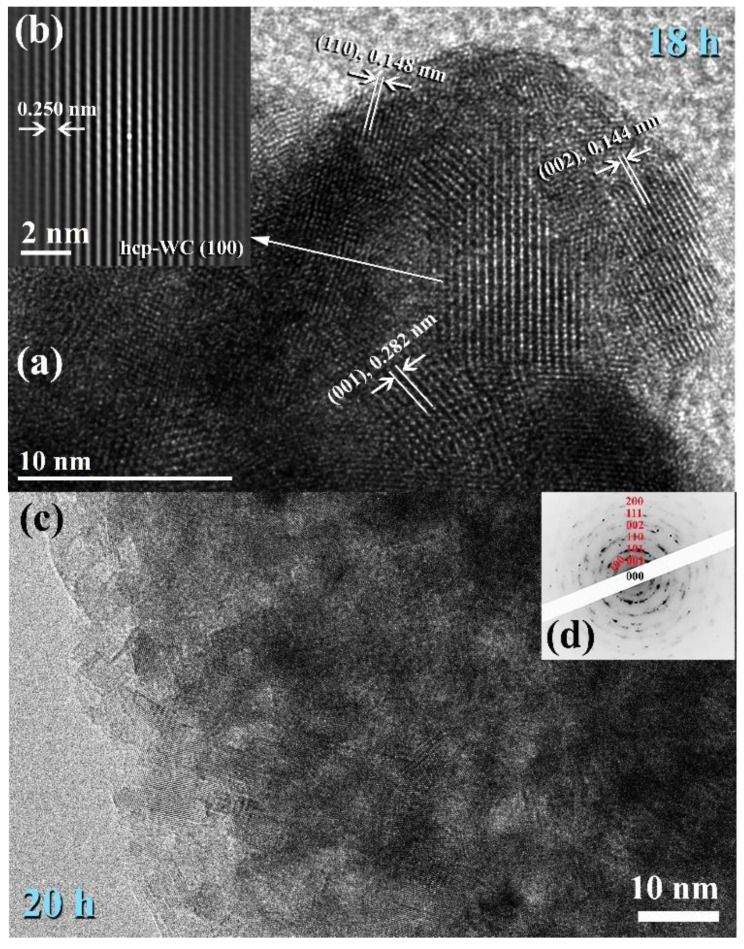
Images of FE-HRTEM for ball milled WC powders and their related selected area diffraction patterns (SADPs) are shown in (**a**,**b**) and (**c**,**d**), respectively.

The FE-SEM micrograph demonstrates that the powders generated after 18 h of MM had a wide range of sizes (50–550 nm) and morphologies (bulky-, rugby ball-, and spherical-like shape), as illustrated in Figure 13a. Following this step of MM, the WC powders shown in Figure 13a consisted of nano-sized grains (5–10 nm) of polycrystalline hcp-WC with various lattice planar orientations ((110), (002), (001), and (100)), as illustrated in Figure 14a,b. Due to a decrease in the atomic level strain, the WC crystals were broken along their grain boundaries into nano-dimensional subgrains (Figure 14a). [51].

At the conclusion of the MM processing time, the powders realized the benefits of the 20-h milling, showing broad Bragg peaks (Figure 11f). This indicates that the MM process for generating WC nanopowders has been completed. Additionally, the intense ball-powders-ball (a) directed toward (0001) is illustrated in (b). In (d), the lattice resolution and NBDP of a specified area in (c) are combined [51].

After 18–20 h of milling, the powders exhibited a nanospherical shape with an apparent diameter varying from 20 to 400 nm, as seen in Figure 13a,b. HRTEM micrograph of WC particles milled for 20 h (Figure 14c) As seen in Figure 14, the ultimate product generated after 20 h of MM had many gain boundaries with unpredictable crystallographic orientations (a, b). We should stress that extending the MM duration to 20 h was required to achieve homogenous WC cells with a narrow grain size dispersion (5–9 nm), as shown in Figure 14c.

#### Consolidation of WC with Spark Plasma Sintering

Consolidation of nanocrystalline WC powders is a key stage in establishing the powders’ physical/mechanical characteristics in a consistent and repeatable way, which is required for the majority of industrial and structural applications. Consolidating ultrafine powders into bulk, dense compacts while preserving nanoscale grain size is a serious challenge. Historically, hot pressing (HP) and hot isostatic pressing (HIP) methods were predominantly employed to consolidate WC powders between 1700 and 2000 °C [47,48,51]. These typical consolidation regimes produced either green compacts (75% of theoretical density) with nanograin structure or dense compacts with microstructured grains. Due to the massive surface area of nanocrystalline material powders, they are extremely unstable and display significant grain development when subjected to high temperatures, such as those employed in HP and HIP. Historically, pure metallic powders like Co, Cr, and Ni have been added to the WC to speed up the sintering process and assure the formation of dense compacts. Unfortunately, the presence of a metallic matrix with such a low melting point has traditionally resulted in a variety of problems, including reduced hardness and corrosion/oxidation resistance, as well as increased grain development [57].

**Figure 15:**
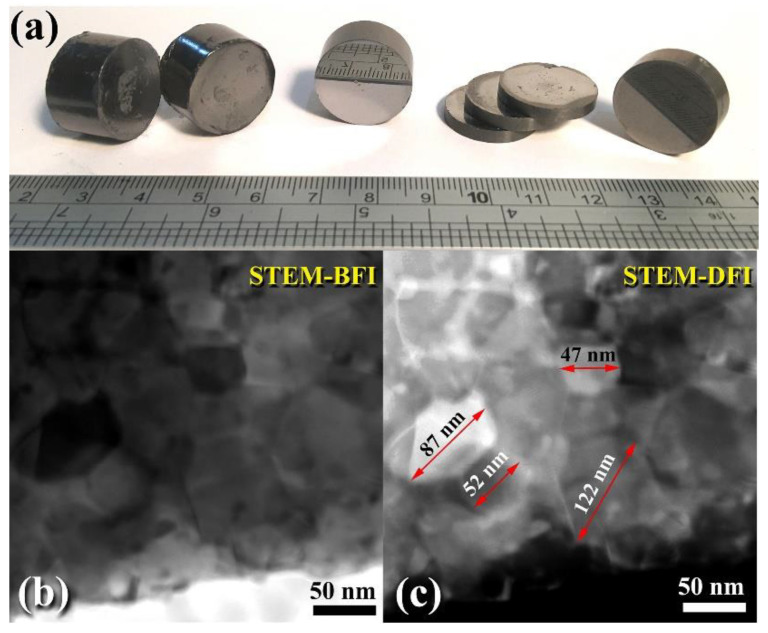
(**a**) A photograph demonstrating the shapes and sizes of various WC consolidated buttons obtained after the powders for the final product were consolidated (20 h) using the SPS method at 1250 °C. The STEM-BFI and STEM-DFI for the chosen button are depicted in (**b**) and (**c**), respectively.

Spark plasma sintering (SPS) has attracted a significant number of researchers from the global societies of nanotechnology, materials science, and powder metallurgy due to its ability to preserve nanostructured nanopowders with low grain development. As a result, the SPS system has been primarily used to consolidate metastable materials (e.g., nanocrystalline powders) that are extremely temperature sensitive. SPS enables ultrafine powders to be sintered and bonded rapidly at lower temperatures than HP/HIP. Sintering is accomplished by electrically charging the spaces between powder particles and effectively applying a high-temperature spark plasma generated during a brief energizing stage, as well as an electro-magnetic field and/or joule heating via a continuous ON-OFF DC pulsed high electric current with a low voltage. The most effective components in the SPS consolidation process are the current effects and high heating rates. Figure 11g depicts the XRD pattern of as-consolidated WC-bulk button (Figure 15a). Clearly, no phase change occurred during the SPS-consolidation stage, as evidenced by the Bragg lines associated with hcp-WC (Figure 11g). By and large, the diffraction lines shown in Figure 11g exhibited significant widening, implying the presence of nanocrystalline WC grains. To assure the development of nanocrystalline bulk materials, an ion-slicer was used to produce a tiny portion of the as-consolidated WC button for TEM analysis. In Figure 15b,c, the STEM-bright field image (BFI) and the STEM-dark field image (DFI) for the as-consolidated bulk WC are displayed together. As-consolidated powders have a dense structure consisting of fine equiaxed grains, as seen in Figure 15b. However, because the SPS process was carried out in a short period of time and at a temperature (1250 °C), which is significantly lower than the temperature required for WC consolidation via hot pressing (above 1650 °C), and at a high heating rate (300 °C/min), the WC grains exhibited moderate grain growth (Figure 15a). The grain diameters of those dense WC buttons ranged from 47 to 122 nm, with an average diameter of 77 nm. By comparing this value to that of as-milled WC powders (7 nm), it is possible to deduce that the SPS method resulted in a modest grain growth of about tenfold when employed to consolidate WC nanocrystalline powders under the circumstances utilized for powder preparation and consolidation.

**Figure 16:**
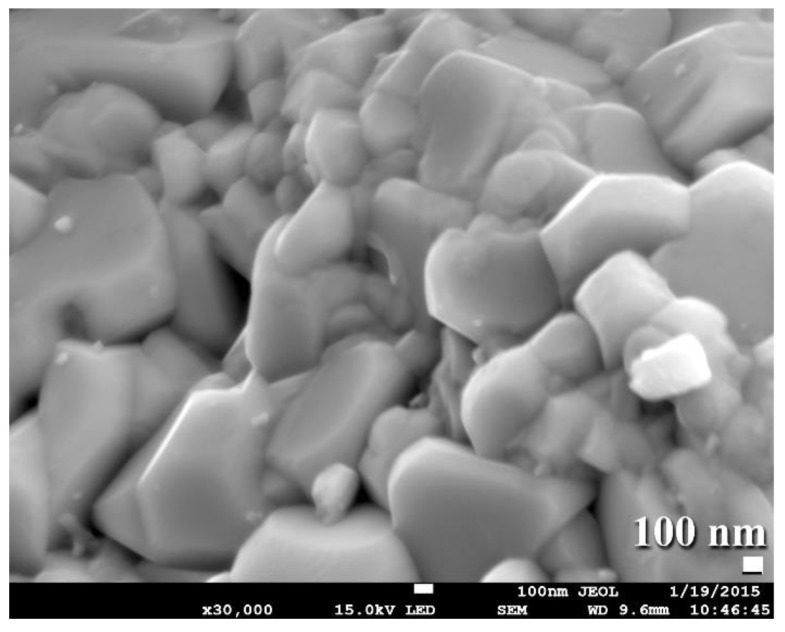
FE-SEM image displays a fracture surface for consolidated WC bulk sample obtained upon SPS the as milled powders for 20 h at 1250 °C.

This grain growth might be a result of the five-minute planned cooling procedure performed to bring the SPS system up to ambient temperature and remove the WC button from the graphite mold. Consolidation of the powders has been attempted at lower temperatures (800–1100 °C). Regrettably, those buttons that consolidated at such low temperatures had extremely low densities ranging from 12.38 to 14.26 g/cm^3^. By comparison, buttons consolidated at 1250 degrees Celsius had a high density of between 15.56 and 15.58 g/cm^3^. Additionally, FE-SEM micrographs of selected buttons solidified at 1250 °C indicate an exceptionally dense nanostructure morphology compatible with the formation of highly dense, fully formed WC prism-shaped fine grains, as seen in Figure 16.

## 4. Nanocomposites

The notion of combining two or more distinct elements to create a new material with superior properties extends all the way back to 3000 BC, when the Egyptian pharaohs discovered that plant fibers could be used to strengthen and prevent cracking of bricks and pottery [1]. The creation of novel composite materials with enhanced physical, chemical, and mechanical characteristics is a key issue of both basic science and applications. The ability to combine different types of materials (metal, ceramic, and nonmetal) to create composite materials offers an excellent opportunity to create new categories of materials with tailored properties by fully exploiting the advantages of both reinforcement and matrix, allowing for a relatively high degree of material design freedom. By combining a compliant isotropic material with low bulk and shear moduli with a stiff isotropic material with high bulk and shear moduli, for example, an elastically isotropic composite with the compliance phase’s bulk modulus and the stiff phase’s shear modulus is formed. The two types of materials that may be found in any composite are matrix and reinforcement (s). Any composite matrix is often more ductile and less stiff, but the reinforcements embedded in it are typically hard and strong [58].

Nanocomposites are materials composed entirely of nanoparticles. [59] refers to a complex family of composite materials made up of at least two phases, one of which is dispersed in the other to create a three-dimensional network. In contrast to conventional composites, at least one of the component phases in nanocomposite materials (either the matrix or the reinforcements) must have a nanoscopic scale dimension [60]. In reality, reinforcements are selected as nanoscaled materials that promote densification of nanocomposites owing to their large surface area and short diffusion distance [61].

### 4.1. Approaches Used for Fabrication of Metal Matrix Composites (MMCs)

MMCs are developed to have a variety of properties, including ductility, toughness, hardness, and stiffness. This can be done by injecting hard ceramic reinforcements into the metal ductile matrix. By combining these two types of materials, new materials with a broad variety of characteristics not seen in single-phase materials can be created. The following summarizes the critical variables affecting the features of MMNCs: (1) particle and grain sizes of reinforcements and matrix; (2) morphological characteristics; (3) volume percentages of reinforcement additions; and (4) reinforcement distributions within the host metallic matrix.

The number of industry-based nanocomposites has risen significantly in recent years. Nanocomposites are used in a broad number of sectors. These new nanotechnology-based materials have a wide range of potential applications in a number of sectors, including medical, electronics, sports equipment, aircraft, and cars. To produce particulate MMNCs, solidifications, the vertex process, squeeze casting, powder metallurgy (PM), hot pressing, and spray and spray deposition are all used. While solidification is believed to be less costly than other manufacturing methods, technical problems emerge throughout the process owing to the propensity of the nano-reinforcement particles introduced to the matrix to agglomerate due to van der Waals force, electrostatic forces, and surface tension. Additionally, the oscillating probe used in the ultrasonic casting process may create significant technical difficulties [61]. As a consequence, if the probe comes into close contact with the liquid metal, it may disintegrate. Inhomogeneous particulate MMNCs with poorly dispersed reinforcing agglomerated nanoparticles embedded in the metal matrix may arise from melting and casting processes.

#### Fabrication of Al/SiC Nanocomposite Powders by Ball Milling

Due to the large thermal expansion coefficient difference between the two constituents, as well as the low wettability of molten Al metal (or Al alloys) and SiC, the traditional technique of liquid metallurgy, which is the least expensive method for composite fabrication, cannot be successfully used to synthesize a large number of nanocomposite systems. When SiC interacts with molten aluminum, Al_4_C_3_ and Si are formed. This reaction is known to have a variety of negative effects on the composite’s overall properties, which can be summarized as follows:

- The mechanical properties of SiC will degrade as a result of Al_4_C_3_ synthesis.
- Additionally, due to the instability of the reaction product Al_4_C_3_ in certain conditions, such as water, methanol, and hydrochloric acid, the composite may be susceptible to corrosive environments.
- Additionally, Si produced as an interfacial reaction product will form Al-Si eutectic at the interface and grain boundary areas, resulting in the composite exhibiting undesirable mechanical properties.

El-Eskandarany [59] produced metal matrix, an Al reinforced with SiC_p_ composite with unusual nanocrystalline characteristics, using a high energy ball mill. He used a sapphire mortar and pestle to weigh pure elemental powders of Al (99.99 percent, 10 mm) and SiC (phase, 99.9 %, 100 mm) and mixed them in a glove box in a refined argon atmosphere. The original powder combination was then charged and sealed with 50 stainless steel balls in a stainless steel vial (SUS 316, 250 mL capacity) (SUS 316, 10 mm in diameter). The ball-to-powder weight ratio was maintained at 20:1. Ball milling experiments were done in a high energy planetary ball mill at a rotating speed of 300 rpm. To avoid the occurrence of any undesirable polluted phases, the ball-milling experiments were halted periodically (every 1.8 ks) and then restarted when the temperature of the vial reached about 300 K. The final product (86 ks of milling) of the mixed powders was then compressed in vacuum for 0.3 ks at a pressure of 19.6–38.2 MPa utilizing a plasma assisted sintering method (PAS).

**Figure 17:**
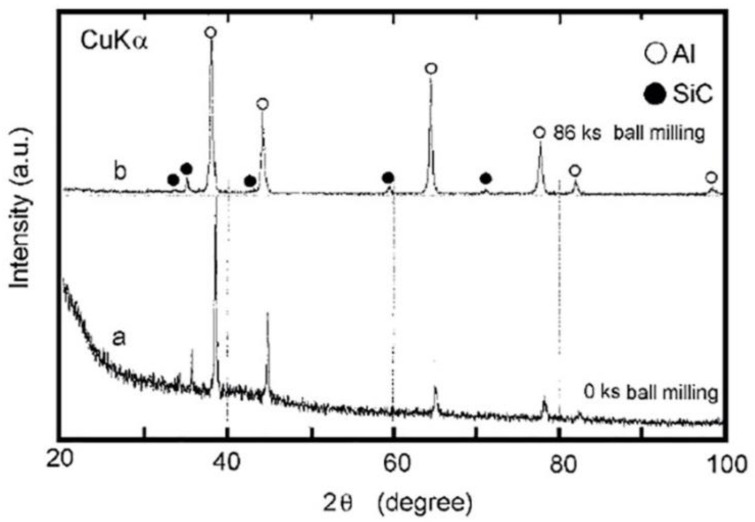
XRD patterns of ball milled SiC_10_/Al_90_ powder after (**a**) 0 ks and (**b**) 86 ks.

The as-milled and as-consolidated materials were examined by XRD with CuK_a_ radiation, SEM with a 20 kV microscope, TEM with a 200 kV microscope, and chemical analyses using induction coupled plasma emission and helium carrier fusion-thermal conductivity, respectively. The contamination levels in the composite SiC_p_/Al final product were determined to be less than 0.07 and 0.2 (at. %) for gas (oxygen, nitrogen, and hydrogen) and iron introduced into samples from milling tools, respectively. Through water immersion, Archimedes’ principle was utilized to determine the density of the consolidated samples. The hardness of the compressed sample was determined using a Vickers indenter loaded with 10 kg. Additionally, the elastic characteristics of the bulk samples were studied utilizing an ultrasonic pulse-echo overlap method and an ultrasonic detector. The typical XRD patterns of mechanically mixed SiC_10_/A1_90_ powder after 0 ks (beginning material mixture of elemental Al and SiC powders) and 86 ks (the end-product) are shown in Figure 17a,b, respectively. Obviously, the starting materials include coarse polycrystalline grains of SiC and Al, indicated by the strong diffraction peaks of the powder combination. On the other hand, as milling progresses, the Bragg peaks of both the reinforcement (SiC) and metallic matrix (Al) powders grow wide (Figure 17b), suggesting the development of a nanocomposite SiCp/Al material.

**Figure 18:**
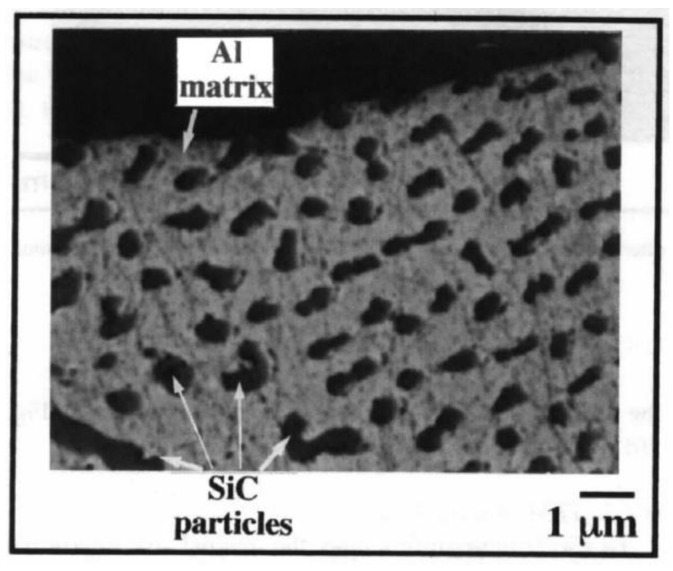
SEM micrograph of the cross-section of a selected ball milled SiC_10_/Al_90_ particle following 43 ks milling.

Surprisingly, this end product remains an integral mixture of polycrystalline SiC and Al and does not coexist with any reactive products such as Al_4_C_3_ or Si, suggesting that no negative reactions occurred at the SiC/Al interfaces. The resulting SiC_p_/Al nanocomposite displayed exceptional morphological characteristics, as evidenced by the homogeneous distribution of SiC inside the Al matrix (Figure 18). This final product contains intact polycrystalline SiC and Al and does not coexist with any reactive products, such as Al_4_C_3_ and Si, suggesting that no negative reactions occurred at the SiC/Al interfaces. The resulting SiC_p_/Al nanocomposite displayed exceptional morphological characteristics, as evidenced by the homogeneous distribution of SiC inside the Al matrix (Figure 18).

**Figure 19:**
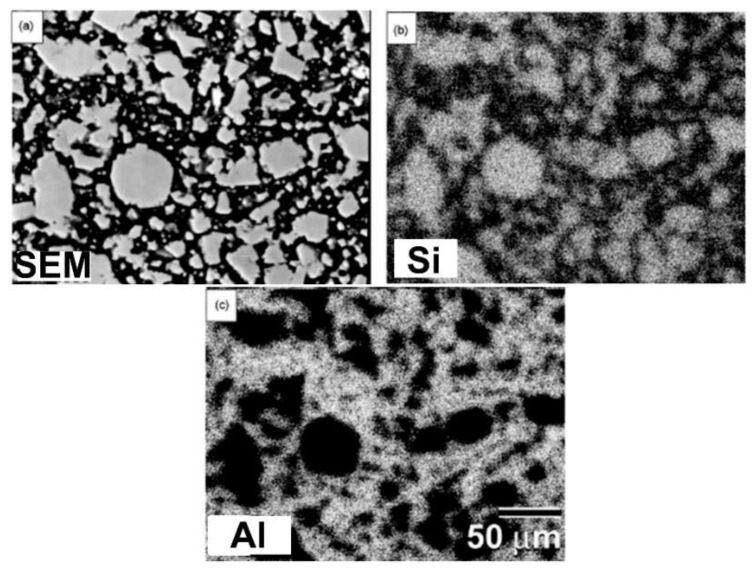
(**a**) SEM micrograph, elemental mapping of (**b**) Si, and (**c**) Al for as-consolidated SiC_10_/Al_90_ that was ball milled for 86 ks.

Figure 19 shows the SEM (back scattering) micrograph and the elemental dot mapping of Al and Si in the as-consolidated SiC_10_/Al_90_ compact. The SiC particles are imbedded and dispersed throughout the Al matrix. There seem to be no cavities or fractures, suggesting a good SiC/Al interfacial bonding (Figure 19a). Furthermore, no amorphous or intermediate phase reaction product can be detected, as indicated by the segregation of Al and Si components in the composite sample on a micron scale, as illustrated in Figure 19b,c.

**Figure 20:**
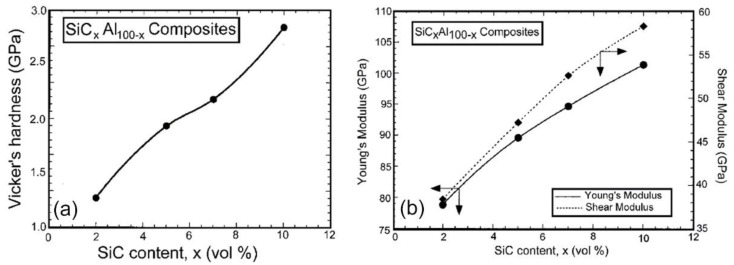
Correlation between the SiC content, x, and the (**a**) Vickers hardness and (**b**) elastic moduli of consolidated SiC_x_/Al_100-x_ powders produced after 86 ks ball milling.

Figure 20a illustrates the effect of the SiC additions on the Vickers hardness of the consolidated samples. As expected, the hardness increases linearly with increasing volume fractions of the harder phase (SiC) in the soft matrix of metallic Al, reaching a maximum of approximately 2.6 GPa for the sample containing 10% SiC, which is significantly greater than the measured hardness of pure Al (about 0.95 GPa) under the same measurement conditions. Surprisingly, the distribution of hardness values across all SiCx/Al100-x samples is narrow, implying a homogeneous distribution of reinforcement particles inside the Al matrix. The bulk modulus, Young’s modulus, and shear modulus of the consolidated mechanically mixed SiC_x_/Al_100-x_ composites are shown in Figure 20b as a function of the SiC content. We should stress that the elastic moduli of the consolidated samples were calculated using the observed sample densities and the non-destructive testing apparatus’s constant parameters. These values substantially rise when the SiC concentration is increased, implying the development of a composite material with a brittle phase (SiC) contained in a ductile Al matrix.

### 4.2. Fabrication of Ceramic Matrix Composites of 100-x(93-WC/7(10Co/4Cr))/x-ZrO_2_ System

**Figure 21:**
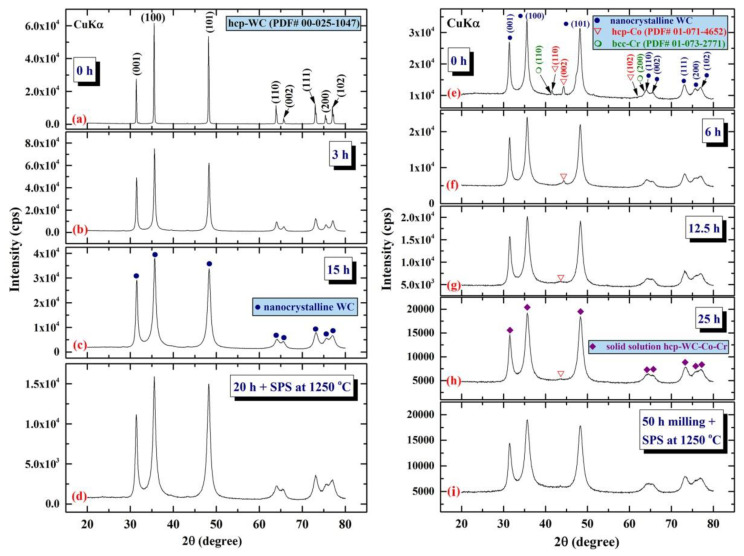
XRD patterns of starting hcp-WC powders after (**a**) 0, (**b**) 3, and (**c**) 15 h mechanical milling. The particles were milled for 20 h and then consolidated at 1250 °C using the SPS method (**d**). The XRD patterns of WC powders that have been ball milled and then mechanically mixed with 7% (10Co/4Cr) powders for 0, 6, 12.5, and 25 h are shown in (**e**–**h**). The XRD pattern of powders containing 93-WC/7 wt. % (10Co/4Cr) produced after 50 h and subsequently consolidated at 1250 °C using the SPS method is given in (**i**).

Cemented carbides [62], for example, tungsten carbide–cobalt composites, include a high percentage of carbide, which confers superior hardness and wear resistance [62]. Due to the uncommon combination of characteristics such as high hardness, mild toughness, and good wear resistance, this class of materials has been extensively used in the manufacturing of cutting tools and wear-resistant components. Due to the high melting point of WC (about 2900 °C), producing dense pure WC in large quantities using standard sintering techniques is very difficult [63,64]. While adding a Co metal binder to WC improves sintering and increases strength and toughness, it considerably decreases the cemented carbides’ hardness and wear resistance. Additionally, composites using such metallic binders perform poorly in corrosion and high temperature applications as compared to WC [65]. All of these properties limit the application of WC-metal binder composites. In 2001, El-Eskandarany [66] produced fully dense bulk nanocomposites with superior mechanical properties by substituting a nonmetal-binding nanocrystalline MgO Al_2_O_3_material for the usual metal Co binder. Since then, nanocrystalline Al_2_O_3_ [67] and ZrO_2_ [68] have been employed as nonmetal binders to improve the mechanical and physical properties of WC materials. As shown in Figure 21a, the first WC powder was polycrystalline with strong Bragg peaks consistent with the hcp-WC phase (PDF# 00-025-1047).

**Figure 22:**
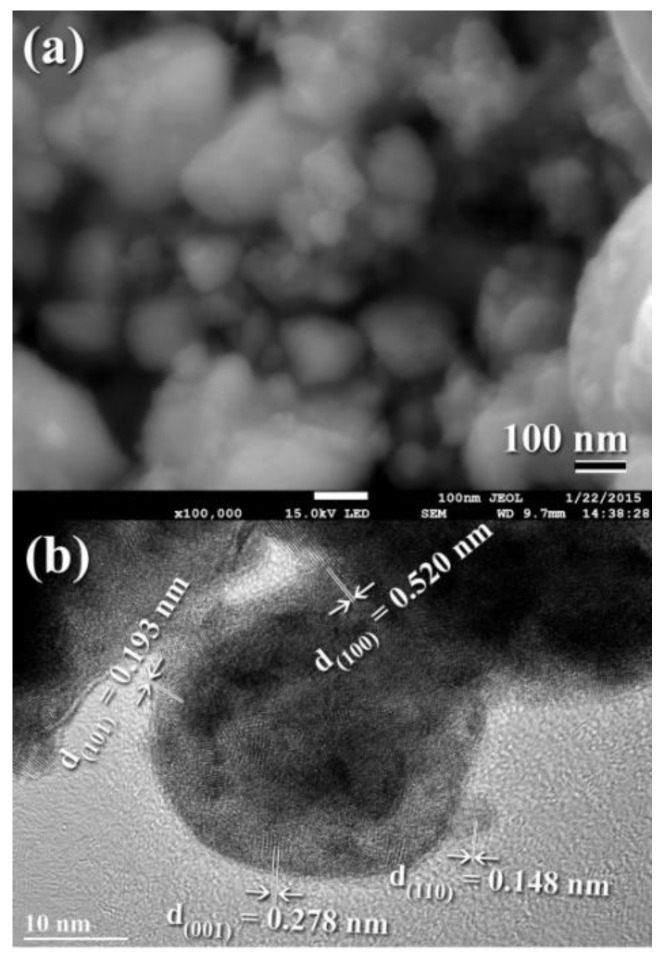
(**a**) FE-SEM, and (**b**) HR-TEM micrographs of mechanically ball milled hcp-WC for 20 h.

When powders are continually ball milled for three hours, significant widening of the main ((001), (100), (101)) and minor ((110), (002), (111), (200), (102) Bragg peaks shows grain refinement caused by the mechanical process (Figure 21b). Figure 22a depicts FE-SEM micrographs of the end product of WC powders produced after 20 h of mechanical milling, while Figure 22b depicts an HR-TEM micrograph of the final product of WC powders. The particles had smooth surfaces and became ultrafine to an apparent diameter of 185 nm on average (Figure 22a). Due to the Van de Walle force, the nanopowders as ball milled tended to agglomerate to form larger particles with an average diameter of 413 nm, as shown in Figure 22a. These nanopowder particles were composed of ultrafine grains ranging in diameter from 3 to 8 nm, as illustrated in Figure 22b. The indexed grains in Figure 22b had different orientations related to interplanar spacing (d) of 0.520, 0.193, 0.278, and 0.148 nm that matched well with (100), (101), (001) and (110), respectively (PDF# 00-025-1047). When nanocrystalline WC powder particles produced after 20 h of MM were consolidated at 1250 °C using the SPS method, the materials retained their nanocrystalline properties, as shown by the widening of the hcp-WC Bragg peaks shown in Figure 21d. This implies that bulk nanocrystalline WC material has formed.

After 20 h, these fine WC nanopowder particles were mixed with powders containing 7% (10Co/4Cr) and ball milled for a variety of MM times. Figure 21e illustrates the XRD pattern of the first WC/7 wt. % (10Co/4Cr) powders (before ball milling, 0 h). The powders contained nanocrystalline hcp-WC (reinforcement materials) and elemental hcp-Co (PDF# 01-071-4652) and bcc-Cr (PDF# 01-073-2771). Following MM for a duration ranging from 6 to 25 h, the elemental metallic powders of Co and Cr were subjected to severe grain refinement produced by the milling medium (WC-balls) and nano-milling media (WC-nanopowders), as indicated by the significantly widened Bragg peaks in Figure 21f.

**Figure 23:**
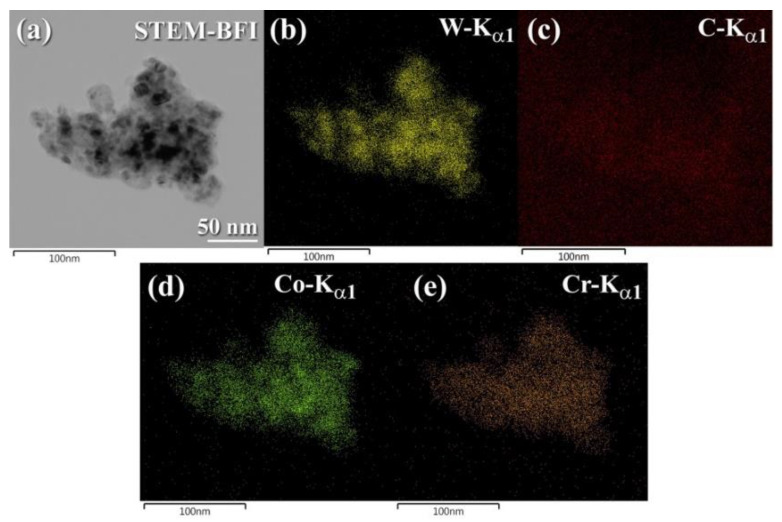
(**a**) STEM-BFI of WC nanocrystalline powders obtained after 20 h of the milling time and then ball milled with 7 wt. % (10Co/4Cr) powders for 50 h, using a high energy ball mill. The corresponding X-ray elemental mapping of W-Kα_1_, C-Kα_1_, Co-Kα_1_, and Cr-Kα_1_ are displayed in (**b**–**e**), respectively.

At the middle (12.5 to 25 h) stage of MM time, the Bragg diffraction patterns associated with the elemental powders of Co and Cr are barely discernible (Figure 21g,h), implying the formation of a solid-solution WC-Co-Cr nanocrystalline phase. To obtain homogeneous WC/7 wt. % (10Co/4Cr) nanocomposite powders, thin WC cells with grain sizes ranging from 18 to 27 nm (Figure 23a) were uniformly embedded (Figure 23b,c) into a thin metallic matrix of Co (Figure 23d) and Cr (Figure 23e). Even after consolidation using the SPS technique at 1250 °C, this homogeneous metastable nanocomposite powder maintained its nanoscaled dimension and nanocrystallinity without displaying substantial grain growth (Figure 21i).

**Figure 24:**
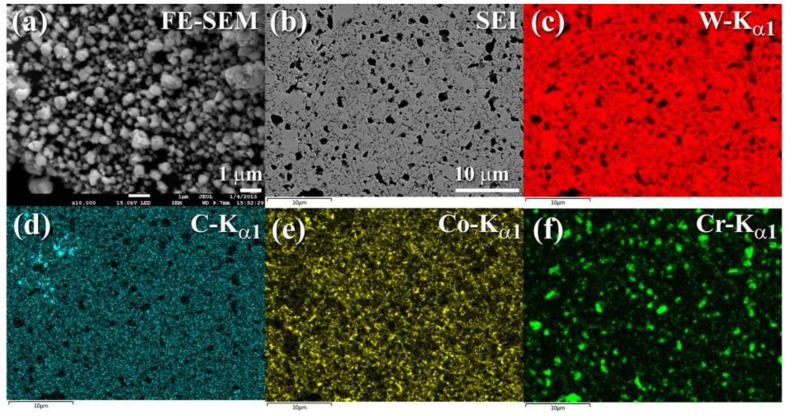
(**a**) FESEM of nanocomposite 93-WC/7 (10Co/4Cr) powders obtained after 50 of ball milling time. The cross-sectional view of the consolidated powders achieved at 1250 °C, using SPS technique is presented in (**b**) together with the corresponding X-ray elemental mapping of (**c**) W-Kα_1_, (**d**) C-Kα_1_, (**e**) Co-Kα_1_, and (**f**) Cr-Kα_1_.

Figure 24a illustrates a FE-SEM micrograph of nanocomposite WC/7 wt. % (10Co/4Cr) powders after 50 h of MM. Following this stage of ball milling, the powders took on a spherical form, with particle diameters ranging from 0.35 to 1.2 mm, as seen in Figure 24a. In Figure 24b, the sample developed small grains following consolidation using SPS at 1250 °C. The SPS-synthesised nanocomposite materials displayed an exceptionally uniform distribution of the acute angle fine-grain (less than 5 mm in diameter) associated with the WC (Figure 24b–d) inside the metallic matrix of Co (Figure 24e and Cr) (Figure 24f). The as-consolidated powders lacked porosity and reinforcement-free zones, indicating the formation of a homogenous bulk WC/6 weight percent (10Co/4Cr) nanocomposite material.

**Figure 25:**
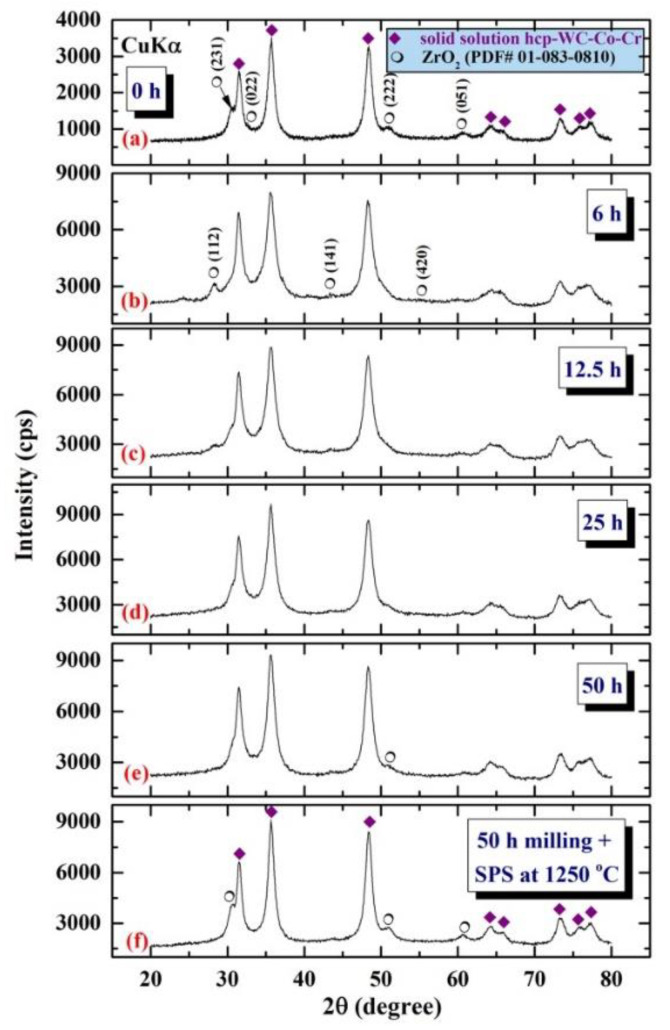
XRD patterns of 93-WC/7 wt. % (10Co/4Cr) powders after 50 h of milling and then mixed with 7 wt. % (ZrO_2_) powders for (**a**) 0, (**b**) 6, (**c**) 12.5, (**d**) 25, and (**e**) 50 h. (**f**) shows the XRD pattern of nanocomposite 93(93-WC/7 wt. % (10Co/4Cr))/7 wt. % ZrO_2_.

After 50 h of milling, the powders of 93-WC/7 wt. % (10Co/4Cr) nanocomposite were mechanically mixed for varied milling times with 7 wt. % (ZrO_2_/1.5 wt. percent Y_2_O_3_). As depicted in Figure 25a, the powders originally consisted of solid-solution hcp-WC-Co-Cr and big crystalline ZrO_2_ grains (PDF# 01-083-0810). When the MM time was increased between 6 and 12.5 h (Figure 25b), those Bragg reflections associated with ZrO_2_ tended to exhibit obvious widening and a decrease in their intensities (Figure 25c). This might indicate that the ZrO_2_ phase has diffused into the hcp-WC-Co-Cr matrix. When the MM time was prolonged to 25 h (Figure 25d) or 50 h (Figure 25e), the Bragg peaks associated with the ZrO2 phase were hardly detectable, implying the production of metastable hcp-93(93-WC/7 wt. percent (10Co/4Cr)) /7 wt. % ZrO_2_ powders.

**Figure 26:**
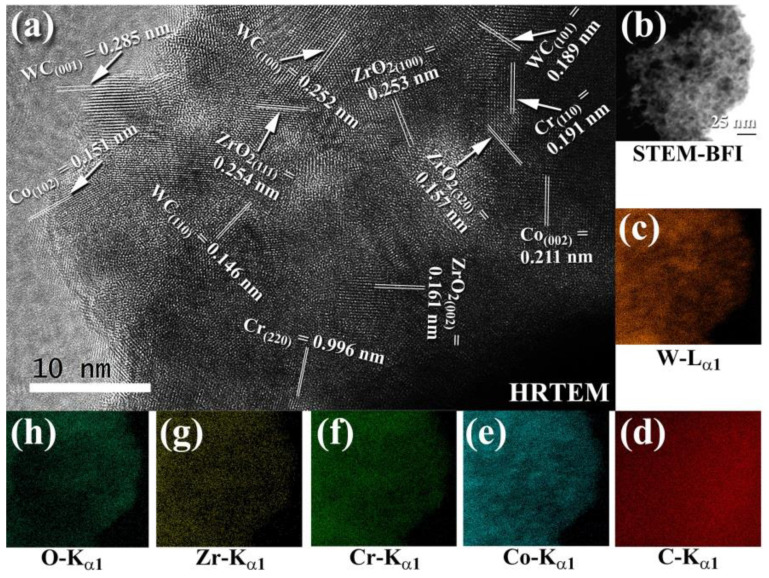
(**a**) HRTEM and (**b**) STEM-BF pictures of nanocomposite powders 93(93-WC/7 wt. % (10Co/4Cr))/7 wt. % ZrO_2_ produced after 50 h of ball milling. The equivalent X-ray elemental mappings for (**c**) W-L_a1_, (**d**) C-K_a1_, (**e**) Co-K_a1_, (**f**) Cr-K_a1_, (**g**) Zr- K_a1_, and (**h**) O-K_a1_ have been established.

The HRTEM image of 93(93-WC/7 wt. % (10Co/4Cr))/7 wt. percent ZrO_2_ powders generated after 50 h of MM is shown in Figure 6a. Numerous small grains (5–10 nm in diameter) of WC, Co, Cr, and ZrO_2_ were found in the sample, as seen in Figure 26a. STEM-BFI analysis (Figure 26b) and X-ray EDS analysis (Figure 26c–h) The ultrafine WC grains (8 to 20 nm in diameter, Figure 26b–d) with lens-like morphology (Figure 26b) were evenly dispersed throughout the light gray matrix of Co (Figure 26e)/Cr (Figure 26f)/ZrO_2_ (Figure 26g,h). After consolidation with SPS at 1250 °C, this metastable solid-solution phase tended to dissociate into hcp-WC-Co-Cr coexisting with ZrO_2_, as shown by the appearance of the Bragg peaks associated with the ZrO_2_ phase in Figure 25f.

**Figure 27:**
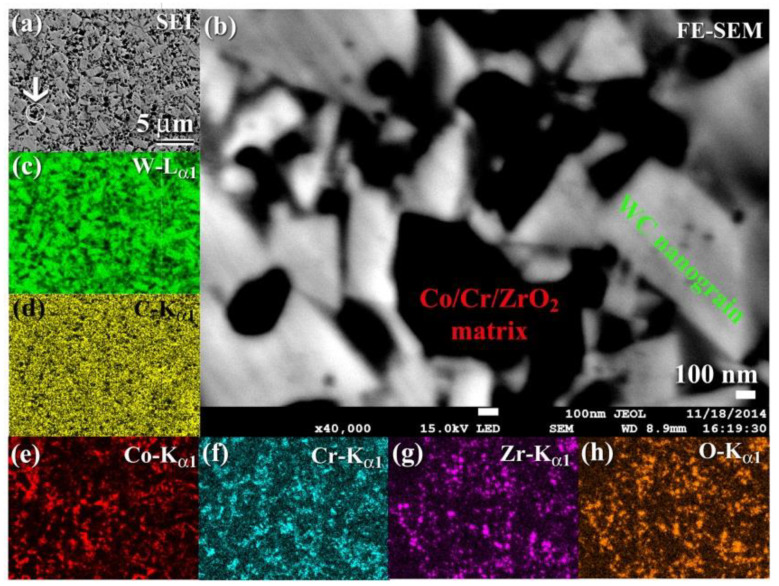
(**a**) FESEM micrograph of the cross-sectional view of as-ball milled nanocomposite 93(93-WC/7 wt. % (10Co/4Cr)) /7 wt. % ZrO_2_ powders (50 h) and then consolidated at 1250 °C, using the SPS technique. The high-magnification (×40,000) for the zone indexed by a circular symbol shown in (**a**) is presented in (**b**). The X-ray elemental mapping corresponding to (**a**) are shown in the figure for (**c**) W-Lα_1_, (**d**) C-Kα_1_, (**e**) Co-Kα_1_, (**f**) Cr-Kα_1_, (**g**) Zr-Kα_1_, and (**h**) O-Kα_1_.

Figure 27a is a FESEM micrograph of the cross-sectional view of 93(93-WC/7 wt. % (10Co/4Cr)) /7 wt. % ZrO_2_ powders after 50 h of MM and then consolidated into bulk material using SPS at 1250 °C. Generally, the composite material had a fine structure, with micro-grained WC embedded in a Co/Cr matrix reinforced with ZrO_2_ (Figure 27a). In Figure 27b, a high-magnification FE-SEM micrograph of the indexed area in Figure 27a is shown. One might claim that the consolidated sample was dense and free of defects or voids. Additionally, the WC grains (light gray grains) did not develop significantly during consolidation, as shown by their modest grain sizes (less than 1 mm in diameter), as illustrated in Figure 27b. It should be noted that the dark matrix zone (Figure 27b) was reinforced by ZrO_2_ fine nanopowder particles (Co (Figure 27c) and Cr (Figure 27f) (Figure 27g,h). The presence of such abrasive ZrO_2_ particles in the metallic matrix may function as grain development inhibitors for WC grains (Figure 27c,d), allowing them to retain their nanocrystalline properties.

**Figure 28:**
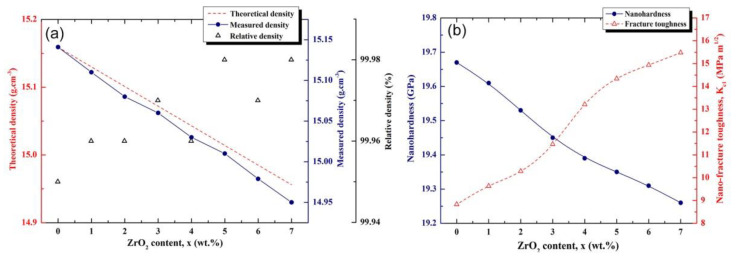
(**a**) Theoretical (broken line), measured (closed symbols), and relative densities (open symbols) of bulk 93(93-WC/7 wt. % (10Co/4Cr)) mixed with various ZrO_2_ concentrations, x. The relationship between nanohardness and fracture toughness and the ZrO_2_ concentration in bulk nanocomposite materials 100-x(93-WC/7(10Co/4Cr))/x ZrO_2_ is shown in (**b**).

For various ZrO_2_ concentrations (x), the bulk densities of the nanocomposite 100-x(93-WC/7(10Co/4Cr))/x ZrO_2_ were computed using the law of mixtures and are presented in Figure 28a alongside the measured and relative densities. All consolidated samples had relative densities more than 99.95 %, suggesting that they underwent a thorough densification treatment that decreased pore fractions. Additionally, when the volume percentage of ZrO_2_ in the nanocomposites grows, the bulk density falls correspondingly. This is a logical consequence of the ZrO_2_ phase taking the place of the high-density WC, metallic Co, and Cr fractions.

Figure 28b illustrates the relationship between nanoindentations and nanofracture toughness and the ZrO_2_ concentration for bulk nanocomposite materials 100-x(93-WC/7(10Co/4Cr))/x ZrO_2_). The findings indicate that increasing the ZrO_2_ content resulted in a small reduction in nanohardness, from 19.67 GPa (for # 93WC/7(10Co/4Cr)) to 19.26 GPa (for # 93WC/7 (10Co/4Cr))/7 ZrO_2_.

One might conclude that increasing the ZrO_2_ content (x) significantly improves fracture toughness, as shown by the concurrent rise in KC_1_ with increasing ZrO_2_ concentration. The bulk nanocomposite 93(93-WC/7 (10Co/4Cr))/7 ZrO_2_ obtained a maximum compressive strength of 15.48 MPa.m^1/2^. This is much more than the predicted value for 93WC/7(10Co/4Cr), 8.83 MPa.m^1/2^.

## 5. Reactive Ball Milling

Despite the use of conventional methods for preparing metal nitrides and hydrides, the reactive ball-milling (RBM) technique, which was first proposed by Calka et al. [69] and El-Eskandarany et al. [70], has been recognized as a crucial tool for fabricating various metallic nitrides and hydrides via room temperature ball milling. In this method, the ball-milling medium subjects the initial metallic powders to significant shear and impact pressures [70,71]. As a result, the powders are subdivided into smaller particles with a greater surface area, resulting in extremely clean or fresh active surfaces on the powders. Due to the mechanically induced reactive milling, the nitrogen was gettered and completely absorbed by the metallic ball-milled powders’ original atomically pristine surfaces, leading them to react similarly to a traditional gas-solid interaction [71].

### Preparations of Titanium Nitride (TiN)

Nitrides possess unique properties that make them suitable for a broad variety of applications. Their hardness, resistance to high temperatures, and electrical and optical characteristics make them indispensable materials in technology. TiN is used in cutting tools, tool coatings, solar control films, and microelectronics applications. It is more resistant to abrasion than alumina and has a very high thermal stability. Additionally, TiN is chemically inert in the vast majority of etching solutions, has a low receptivity, and serves as an excellent metal diffusion barrier.

The methods of activated reactive evaporation [72], self-propagating combustion [73], high nitrogen pressure (105 atm) and high temperature (1200 °C) are often used to produce the cubic form of TiN. For the synthesis of TiN, a chemical vapor deposition method was employed, including the interaction of titanium tetrachloride (TiCl_4_) with ammonia (HN_3_) at temperatures more than 1000 °C. The preparation is often carried out using low-temperature physical vapor deposition (LT-PVD), atmospheric pressure chemical vapor deposition (CVD), as well as reactive sputtering [74]. The drawbacks of these techniques include the high cost of preparation, the use of hazardous precursor gases (e.g., TiCl_4_), toxic exhaust gases (e.g., HCl, Cl), and an excess of contamination in the final product.

In contrast to the above-mentioned preparation methods, the initial ingredients were pure elemental Ti powder and refined nitrogen gas. Twenty-five stainless steel (SUS 316) balls (10 mm in diameter) were placed into a stainless steel (SUS 316) vial (250 mL) and sealed in a glove box in a purified argon (99.99 wt. %) atmosphere. The ball was 10 times heavier than the powder. For reactive milling, the vial was put in a high energy ball mill equipped with a rotary pump and a gas flow system [70,71].

**Figure 29:**
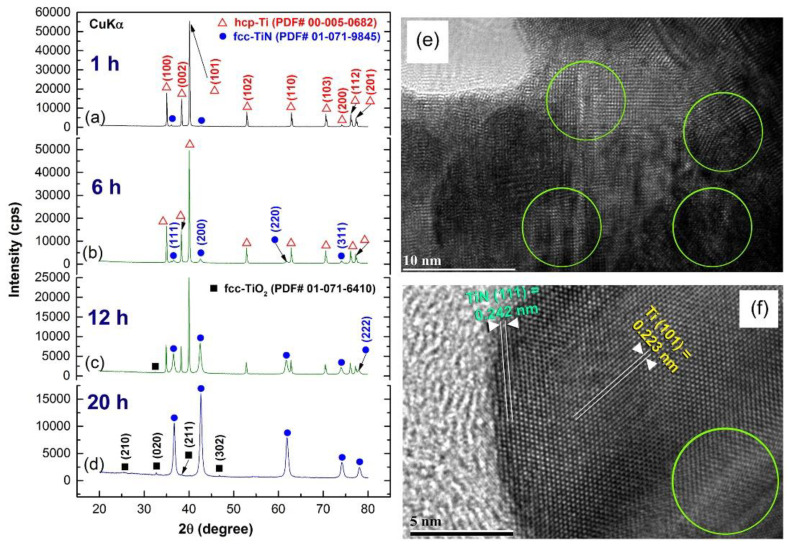
XRD diffraction patterns of Ti powder samples after (**a**) 1 h, (**b**) 6 h, (**c**) 12 h, and (**d**) 20 h of RBM. The HRTEM images of samples milled for 1 h and 3 h, respectively, are presented in (**e**,**f**).

The XRD patterns of the initial elemental hcp-Ti powder samples milled under 10 bar of nitrogen gas for different RBM durations are shown in Figure 29a–d. The powder produced after 1 h included massive crystallites, as shown by the significantly smaller Bragg peaks corresponding to hcp-Ti in Figure 29a and their Miller indices (100), (002), (101), and (110). After this early stage of RBM time, a small mole fraction of the TiN phase was discovered, as shown by the extremely faint Bragg peaks associated with fcc-TiN (#PDF file 01-071-9845), as shown in Figure 29a. At this stage of milling, the powder samples included lattice defects, as shown by the nanotwins, as well as stuck faults inside their internal structure (Figure 29e).

**Figure 30:**
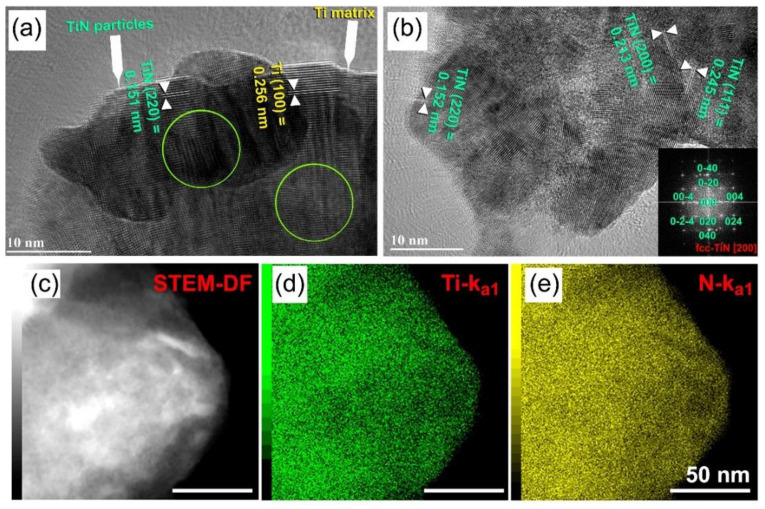
HRTEM image of samples after (**a**) 6 h, and (**b**) 20 h. The FFT of the circle chosen in (**b**) is presented as an inset of (**b**). STEM-DFI images of the powders produced after 20 h of RBM are presented in (**c**), while the matching EDS maps of elemental Ti and N are shown in (**d**) and (**e**).

As seen in Figure 29f, a HRTEM micrograph obtained at the near edge of an aggregated powder particle following 3 h of RBM revealed the existence of a reacted TiN phase embedded in a Ti matrix. Throughout the intermediate period of RBM (6 to 12 h), the Bragg peaks corresponding to the TiN phase grew more pronounced (Figure 29b,c), suggesting a rise in the mole fraction of the reacted TiN phase. The HRTEM image of the powder obtained after 6 h of RBM time reveals two TiN particles with a diameter of about 20 nm on the unprocessed Ti matrix zone (Figure 30a). As seen by the circular symbols in Figure 30a, both the particles and matrix exhibit significant lattice defects, suggesting the occurrence of stacking faults and dislocations. As the RBM time (12 h) increased, the intensity of the Bragg peaks of unreacted hcp-Ti decreased, indicating a reduction in the mole fraction of Ti metal, as seen in Figure 29c.

After 20 h of RBM, all Bragg peaks related with raw metallic Ti particles vanished and a single phase of nanocrystalline TiN with broad Bragg peaks emerged, as seen in Figure 29d. It is worth noting that the Bragg peaks corresponding to the fcc-TiO_2_ phase were generated during the XRD sample preparation procedure as a result of local oxidation of TiN powder surfaces. The lattice parameter a0 of the TiN phase formed after 20 h (end-product) was determined to be 0.423 nm using (111) and (200) reflections, which fits well with the reported value for pure TiN (0.4235 nm, PDF# 01-071-9845). Figure 30b shows a HRTEM micrograph of a TiN powder sample acquired after 20 h of RBM, coupled with fast Fourier transforms (FFT). As seen in Figure 30b, the powder was made of extremely small grains with a diameter of around 8 nm (or less) and a variety of crystallographic orientations (Figure 30b).

EDS was used to analyze the homogeneity of the TiN powders obtained after 20 h of RBM time using the HRTEM/STEM technique. Figure 30c shows a STEM-dark field (DF) image of the near edge of aggregated TiN particles. Based on the related EDS elemental mapping of Ti (Figure 30d) and N (Figure 30e), it can be inferred that both elements were homogeneously distributed throughout the investigated region (100–150 nm) with no elemental segregation beyond the nanoscale level, as shown in Figure 30d (Ti-K_a1_), and Figure 30e (N-K_a1_). Furthermore, the EDS spot analysis of 36 distinct zones, indicated that the average composition (in at. %) of this end-product was Ti_51.68_N_48.32_.

**Figure 31:**
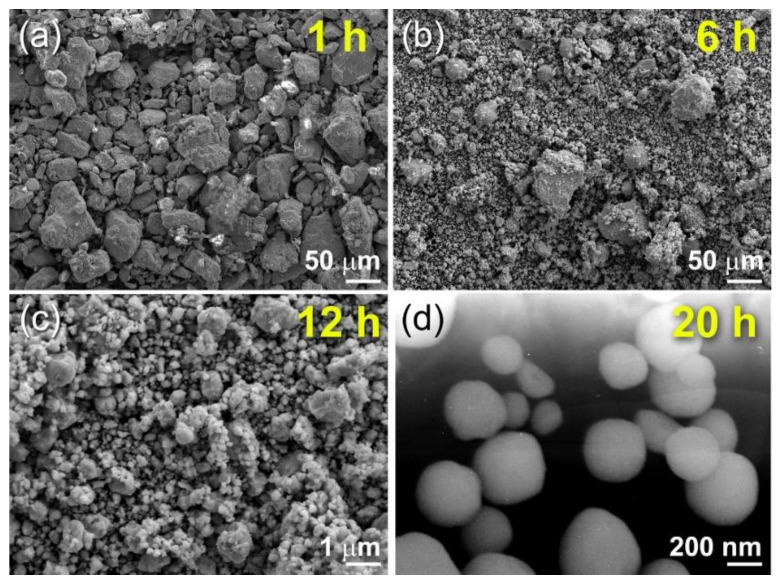
FE-SEM micrograph of the powder samples obtained after (**a**) 1 h, (**b**) 6 h, (**c**) 12 h, and (**d**) 20 h of RBM.

Figure 31a shows FE-SEM micrographs of powder samples collected at the early, middle, and end stages of RBM. As seen in this picture, the sample obtained after one hour of milling exhibited a bulky appearance with particle sizes ranging from 20 to 110 mm. As seen in Figure 31b, extending the RBM duration to 6 h resulted in a considerable reduction in particle size and the formation of significant proportions of large volume spherical particles. Although the big particles in Figure 31b have a low N concentration, the spherical tiny particles (sub 10 mm) have almost equiatomic TiN compositions.

**Figure 32:**
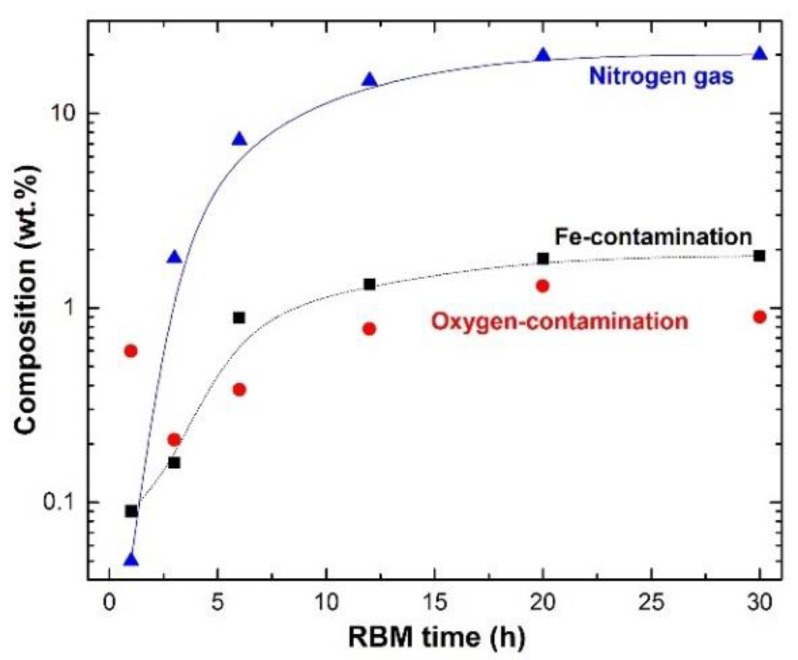
Nitrogen, oxygen, and Fe contents on the milled powder samples obtained after different stages of RBM time.

As demonstrated in Figure 31c, increasing the RBM time to 12 h resulted in a higher percentage of fine TiN particles (less than 1 mm in diameter) compared to untreated Ti powders (1 to 1.7 mm in diameter). The end-product TiN powders had very smooth surfaces and spherical morphology with narrow particle size distribution in the range of 190 to 400 nm in diameter, as illustrated in Figure 31d after the RBM processing period (20 h). Chemical analysis was used to evaluate the nitrogen, oxygen, and iron concentrations of ball-milled powder samples produced at various phases of RBM. The N_2_ content rose proportionally with increasing RBM duration, reaching approximately 18 wt. % after 12 h of RBM (Figure 32). As seen in Figure 32, the Ti powder absorbed 19.7 % of the N_2_ after 20 h of RBM and remained at this level even after a longer RBM duration (30 h). This concludes the gas-solid interaction and results in the production of TiN via the RBM process. As seen in Figure 32, the O_2_ concentration of the powders varies independently of the RBM period. The O_2_ contamination was attributed to improper handling of the powder during sample preparation for chemical analysis outside of the glove box. As shown in Figure 32, the final product produced after 20 h of RBM included approximately 1.4 wt. % O_2_.

To prevent the incorporation of Fe into the powders during the RBM process, the milling tools were coated with Ti metal. Despite this, a trace of Fe (0.09 to 0.85 weight %) was introduced to the Ti powders during the initial stages of milling (3 to 6 h), as seen in Figure 32. Extending the RBM period to 12–20 h raised the volume percent of TiN (hard phase), resulting in an increase in Fe contamination due to ball abrasion. After 20 h, the maximum amount of Fe contamination in the TiN powders was about 2%, as shown in Figure 32.

**Figure 33:**
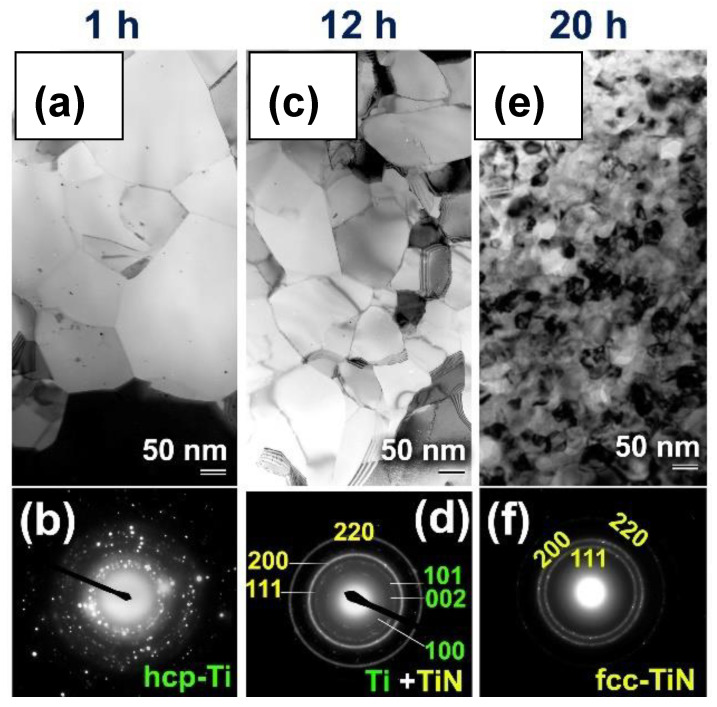
BFI and corresponding SADP of the planar view for as-consolidated and then ion-sliced powder samples obtained after (**a**,**b**) 1 h, (**c**,**d**) 12 h, and (**e**,**f**) 20 h of RBM.

Figure 33 shows bright field imaging (BFI) of ion-sliced SPS-consolidated powder samples after 1, 12, and 20 h of RBM. As confirmed by the corresponding selected area diffraction pattern (SADP) in Figure 33a, the bulk sample of powder milled for 1 h contained large polycrystalline hcp-Ti grains (300 nm in diameter).

The particles were composited with unreacted Ti and fully reacted TiN fine powders after 12 h of RBM (Figure 33b). In this picture, the bulk consolidated sample corresponding to these powders displayed a wide particle size dispersion (50 to 280 nm in diameter). SADP from the center validates the occurrence of the Ti and TiN phases, as seen in Figure 33b. Consolidating the end-product TiN powders into a bulk sample after 20 h of RBM did not result in substantial grain development, as evidenced by the fine nanocrystalline grains with diameters ranging from 42 to 86 nm (Figure 33f). When the grain size of the consolidated bulk TiN compact is compared to the starting size acquired after 20 h of RBM (8 nm), it is evident that the SPS can result in just a small increase of TiN grains of approximately five times.

**Figure 34:**
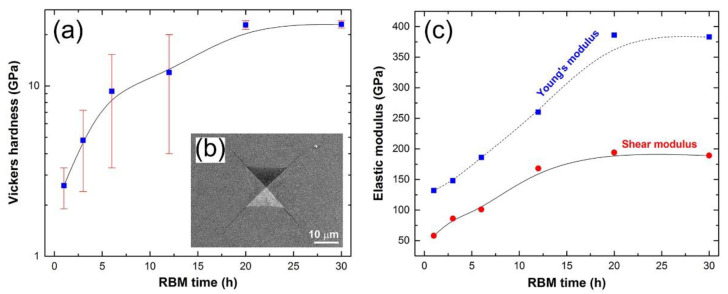
RBM duration has an effect on (**a**) Vickers hardness and (**c**) Young’s and shear modulus. The FE-SEM micrograph of the Vickers indentation fracture surface obtained after 20 h of RBM is shown in (**b**).

Vickers hardness values are presented in Figure 34a for six consolidated independent samples obtained after various milling periods. The figure indicates that the hardness of samples collected during the first stage of RBM (1 to 3 h) ranged between 1.9 and 7.2 GPa. When the RBM time was increased, the volume percentage of unreacted Ti metal in the hard TiN phase increased. The hardness values, which ranged between 4 and 20.2 GPa, attest to this (see Figure 34a). As demonstrated in Figure 34a, the homogenized samples’ hardness values were almost saturated at an average of 22.9 GPa 1.25 near the end of the RBM period (20 to 30 h). This results in the formation of a single TiN phase and the disappearance of Ti metal. The Vickers indentation for the SPSed sample after 20 h of RBM is shown in Figure 34b using a FE-SEM image. The micrograph clearly shows cracks radiating from the indentation edges, indicating that the TiN phase produced is brittle.

The elastic modulus values were calculated using the sample densities obtained and the constant parameters of the nondestructive testing equipment. They were quantified using the Youngs and shear moduli of consolidated samples produced following a variety of RBM durations. The effect of RBM duration on the Youngs and shear moduli is seen in Figure 34c. These values increased significantly throughout the early and middle periods of RBM (1 to 12 h), indicating that the TiN phase in the milled powder increased. At the conclusion of the RBM period (20 to 30 h), both Youngs and shear values are almost saturated at 384 and 192 GPa, respectively, implying the formation of a single homogeneous TiN phase.

## 6. The Drawbacks of Ball Milling and the Subsequent Solutions

### Excessive of Operating Temperature

As with any other process, ball milling has a number of drawbacks that limit the potential to deliver a high-quality end product. Among the parameters that influence the milling process (Section 2.3.3), milling temperature is regarded as one of the most critical variables to control. When high energy ball mills are used, the temperature of the milling tools is often increased. Many researchers in the 1990s used to halt the process for a certain period of time in order to lower the milling temperature. In practice, this technique replicates the time required to manufacture the materials. This issue is viewed as a significant disadvantage of ball milling, particularly when the feedstock is temperature sensitive (e.g., nanocrystalline powders, rubber, or plastics) or extremely soft (certain metals, and polymers) materials.

**Figure 35:**
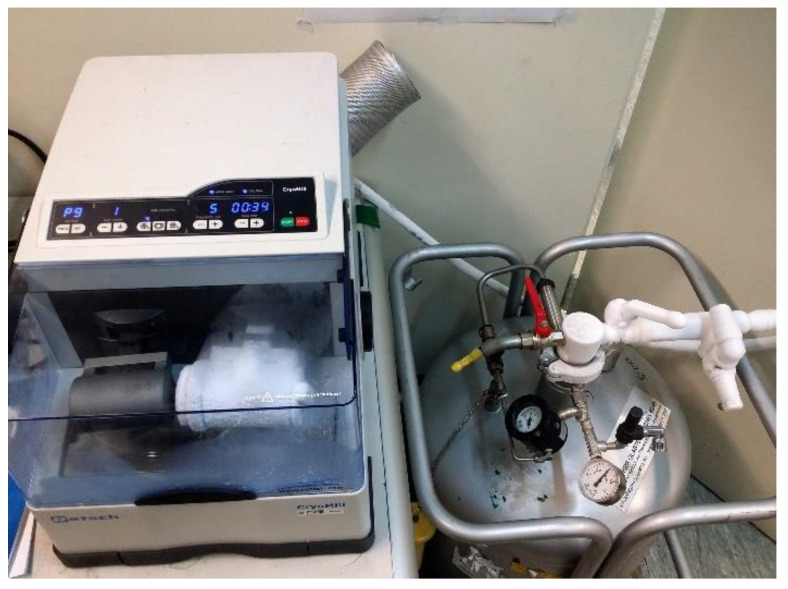
A Cryo-milling equipment housed at NAP/EBRC-KISR.

At the turn of the century, several ball mill manufacturers started to produce cryo-ball mills, which grind the desired materials under a flow of liquid nitrogen. It is designed specifically for cryogenic grinding and crushing of tough or temperature-sensitive materials cooled to −196 °C. Figure 35 displays a photo of a typical cryo-mill produced by Retsch and housed in NAP/EBTC-KISR. The liquid nitrogen is delivered continuously via an autofill system in the precise amount necessary to maintain the temperature at −196 °C. The CryoMill’s grinding jar oscillates radially in a horizontal position, as shown in Figure 35. Due to the inertia of the grinding balls, they impact and pulverize the sample material at the rounded ends of the jar. Recently, cryo-milling has been successfully employed for fabrication of a wide range of materials such as nanoparticles, nanocrystalline and metallic glassy materials [75,76,77,78,79,80,81].

## 7. Conclusions

Mechanical milling (MM) has attracted great attention as a powerful tool for the synthesis of a variety of sophisticated materials, including equilibrium, nonequilibrium (e.g., amorphous, quasicrystals, nanodiamonds, carbon nanotubes, nanocrystalline powders), and nanocomposite materials. The MM is a unique process in that it involves a solid-state interaction between the reactant materials’ fresh powder surfaces at room temperature. As a result, it has been used to fabricate alloys and compounds that are difficult or impossible to acquire using standard melting and casting processes. We have tried to provide some exemplary instances of chosen systems developed at the Kuwait Institute for Scientific Research in this review paper. A broad explanation of the process has been provided, as well as the required feedstock materials, the equipment, and the factors impacting the milling process. Additionally, a brief history of ball milling’s use in the production of a broad range of sophisticated and novel materials during the previous 50 years was provided. Additionally, this article discussed some of the nanocrystalline powders that have been synthesized using MM throughout the previous three decades, including nanodiamonds, nanocrystalline hard materials (e.g., WC), and metal-matric and ceramic matrix nanocomposites. Additionally, the mechanism of mechanically driven gas-solid reaction has been described and addressed in relation to the production of metal nitrides. The authors believe that the paper will be beneficial to readers and will serve as an introduction for engineers and researchers embarking on initiatives involving material fabrication via mechanical milling.

## Data Availability

Not available.
